# Robust Phylogeny of *Tetrastigma* (Vitaceae) Based on Ten Plastid DNA Regions: Implications for Infrageneric Classification and Seed Character Evolution

**DOI:** 10.3389/fpls.2017.00590

**Published:** 2017-04-26

**Authors:** Sadaf Habib, Viet-Cuong Dang, Stefanie M. Ickert-Bond, Jin-Long Zhang, Li-Min Lu, Jun Wen, Zhi-Duan Chen

**Affiliations:** ^1^State Key Laboratory of Systematic and Evolutionary Botany, Institute of Botany, Chinese Academy of SciencesBeijing, China; ^2^University of Chinese Academy of SciencesBeijing, China; ^3^Sino-African Joint Research Center, Chinese Academy of SciencesWuhan, China; ^4^UA Museum of the North Herbarium and Department of Biology and Wildlife, University of Alaska FairbanksFairbanks, AK, USA; ^5^Flora Conservation Department, Kadoorie Farm and Botanic GardenHong Kong, China; ^6^Department of Botany, National Museum of Natural History, Smithsonian InstitutionWashington, DC, USA

**Keywords:** character evolution, classification, molecular phylogeny, seed morphology, *Tetrastigma*, Vitaceae

## Abstract

*Tetrastigma* (Miq.) Planch. is one of the most species-rich genera of the economically and agronomically important grape family Vitaceae. It includes ca. 95 species widely distributed in the tropics and subtropics of Asia and Australia. Species of *Tetrastigma* exhibit great diversity in both vegetative and reproductive characters. Here we inferred a well-supported phylogeny of *Tetrastigma* based on ten chloroplast DNA regions with an expanded taxon sampling of 72 species and two varieties. Our molecular results support six major clades within *Tetrastigma* and the relationships among these clades were well-resolved. We also documented seed morphology of 44 species covering the six major clades of the genus. Ancestral states of eight characters (seed shape, seed surface rumination pattern, chalaza length/width ratio, chalaza position, ventral infold position, ventral infold divergence, ventral infold depth in cross section, and endosperm shape) were reconstructed in Mesquite and R with four models. Character optimizations suggest that all character states have evolved multiple times except that the irregular-shaped surface rumination has derived only once in *Tetrastigma*. We evaluated the taxonomic importance of seed morphology and identified potential morphological evidence to support each major clade. Our comprehensive analyses of *Tetrastigma* shed insights into the infrageneric classification of this morphologically diverse and ecologically important genus in tropical and subtropical Asia.

## Introduction

**Figure 1:**
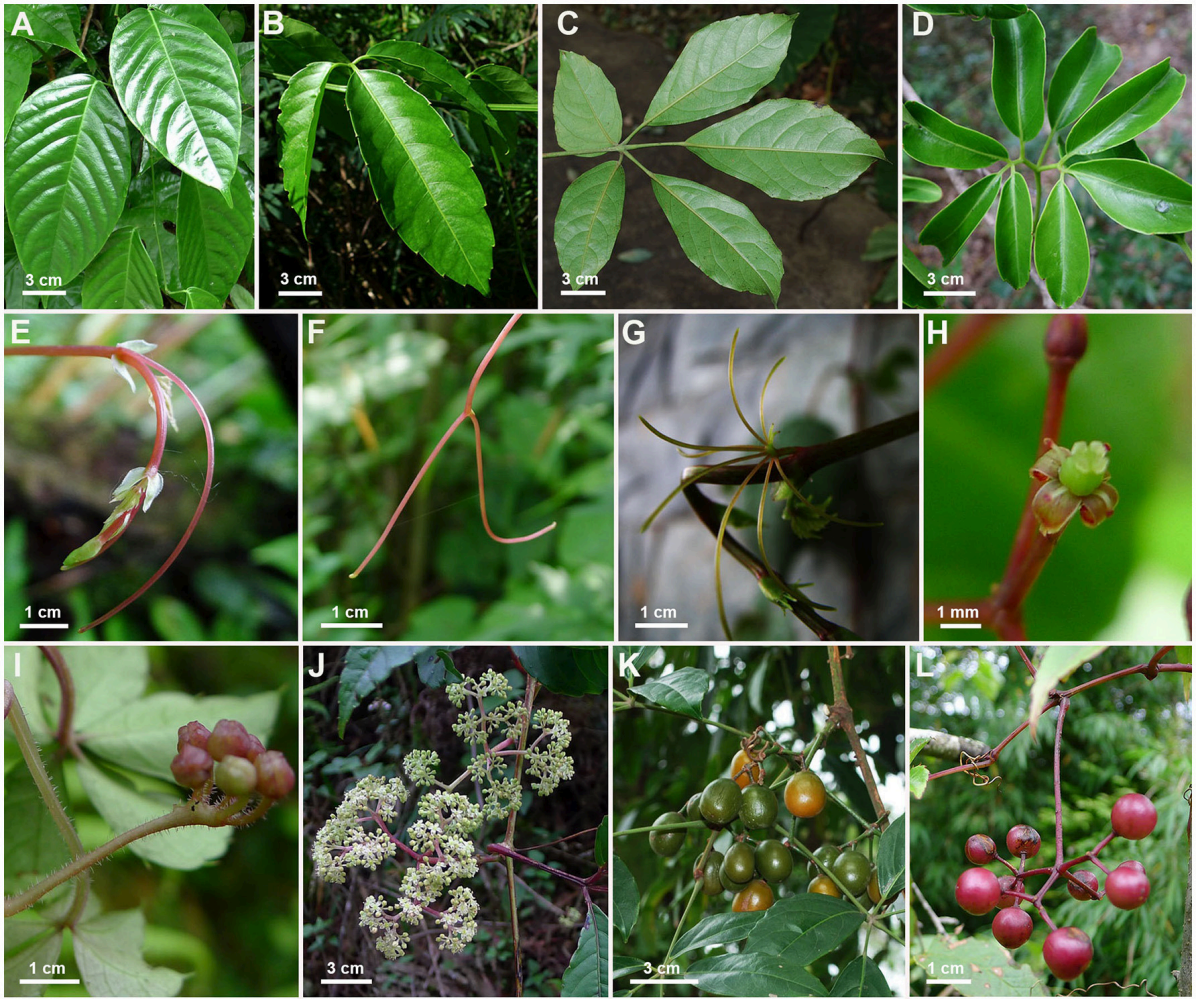
**Morphological diversity in *Tetrastigma***. Leaves: **(A)** Simple; **(B)** 3-foliolate; **(C)** digitately palmate, 5-foliolate; **(D)** pedately palmate, 5–7–9-foliolate. Tendril: **(E)** unbranched; **(F)** bifurcate; **(G)** digitate, 5–7 branched. Flower: **(H)** female, 4-petaled, 4-lobed stigma. Inflorescence: **(I)** simple umbel; **(J)** compound umbelliform. Fruit: **(K)** ellipsoid, **(L)** spheroid. Photographs: **(A–J,L)** photographed by L.M. Lu; **(K)** photographed by B. Liu.

*Tetrastigma* (Miq.) Planch. of Vitaceae (the grape family) contains ca. 95 species of climbers, which are predominantly distributed in the Asian tropics and subtropics with a few species extending to Australia (Süssenguth, 1953; Wen, 2007; Chen et al., 2011a,b). The grape family is of great economical and agronomical importance, containing the grape genus *Vitis* L., and is morphologically unique in having leaf-opposed tendrils and inflorescences (Zhang et al., 2015a). The genus *Tetrastigma* has also attracted a great deal of attention due to its unique host-parasite association with Rafflesiaceae, a family of three genera, which are parasitic exclusively on *Tetrastigma* species and contain the largest flower in the world (Davis and Wurdack, 2004; Davis et al., 2007; Barcelona et al., 2009; Chen et al., 2011a; Nikolov et al., 2014; Pelser et al., 2016). Plants of *Tetrastigma* can dominate diverse habitats, such as tropical rainforests, shrub lands, and limestone mountains (Latiff, 1983; Jackes, 1989; Chen et al., 2007). *Tetrastigma* can be distinguished from other genera of Vitaceae by its tetramerous flowers with distinct 4-lobed stigmas. However, it is difficult to identify *Tetrastigma* specimens to species due to their tremendous morphological diversity in both vegetative and reproductive characters (Figure 1). Furthermore, samples collected in the field usually lack flowers and fruits because reproductive parts of *Tetrastigma* are often only present on the top of the dense canopy of large trees upon which they climb (Pelser et al., 2016), making it more difficult for taxonomical studies.

Within angiosperms, vitaceous seeds are unique and characterized by a dorsal chalaza and a pair of ventral infolds (Tiffney and Barghoorn, 1976; Supplementary Figure 1A). There have been some studies comparing seed morphological characters between *Tetrastigma* and other genera of Vitaceae and seed morphology is considered significant for the infrageneric classification of the genus (Chen and Manchester, 2007; Manchester et al., 2013). The taxonomic importance of seed morphology was further emphasized by Chen (2009) based on morphometric analyses of extant vitaceous seeds, including seven *Tetrastigma* species and highlighting the morphological diversity of *Tetrastigma* seeds (Supplementary Figures 1B–K). Seed morphology of extant vitaceous genera is useful for paleobotanical studies (Chen and Manchester, 2007; Manchester et al., 2013) due to their unique and distinctive morphological features, which are often preserved in fossils (dorsal chalaza and paired ventral infolds). Seed fossils of Vitaceae are usually identified to genus based on their resemblance to extant seeds. There are a few (about 13) macrofossils reported to be *Tetrastigma* (Chandler, 1925, 1961a,b, 1962; Reid and Chandler, 1933; Kirchheimer, 1938; Miki, 1956; Teodoridis, 2003). However, Chen and Manchester (2007) transferred two of them (*Tetrastigma lobatum* Chandler and *T. chandleri* Kirchheimer) to *Ampelocissus* Planch. and questioned the reliability of the remaining fossil taxa as *Tetrastigma*. Thus, a detailed morphological study of modern *Tetrastigma* seeds with expanded taxon sampling will not only provide insights into the classification, but also shed light on the evaluation and confirmation of *Tetrastigma* fossils.

Two well-known taxonomic classifications of *Tetrastigma* are from the species-rich regions of China and the Malay Peninsula (Latiff, 1983; Li and Wu, 1995). Based on fruit and seed morphology, Latiff (1983) divided the 12 species from the Malay Peninsula into two sections: section *Carinata* Latiff and section *Tetrastigma*. Section *Carinata* (3 species) is characterized by pyriform and 3–4 seeded berries, obtriangular seeds with chalaza extending to the middle of the dorsal surface, diverging ventral infolds, and T-shaped endosperm in cross section. Section *Tetrastigma* (9 species) possesses globose to ellipsoid and 1–2-seeded berries, globose to obovoid-elliptic seeds with chalaza extending ¾ ways or more of the seed length, and endosperm M- or T-shaped in cross section. By studying 42 species from China, Li and Wu (1995) recognized the branching pattern of tendrils and inflorescence architecture as important taxonomic characters for the infrageneric division of *Tetrastigma* in addition to seed morphology. This study provided an infrageneric classification of the genus with two subgenera: subgenus *Palmicirrata* C.L. Li and subgenus *Tetrastigma*. Subgenus *Palmicirrata* (3 spp.) is characterized by digitately-branched tendrils and compound cymes, while subgenus *Tetrastigma* (39 spp.) has unbranched or biforked tendrils and inflorescences that are usually polychasia. Moreover, Li and Wu (1995) accepted the circumscription of sections proposed by Latiff (1983) and described a new section, *Orbicularia* C.L. Li., on the basis of rugulose or tuberculate seeds with a rounded to elliptic chalaza in the middle of the dorsal surface of the seed. There have been several other regional studies concerning the taxonomy of *Tetrastigma* (Gagnepain, 1910, 1944; Merrill, 1912, 1916, 1918; Craib, 1931). However, the significance of seed morphology in *Tetrastigma* taxonomy has not been thoroughly examined in these studies. Thus, there has been no worldwide classification of *Tetrastigma* and the infrageneric classification status of some species remains unknown.

The monophyly of *Tetrastigma* has been supported by previous molecular studies of Vitaceae with limited sampling of *Tetrastigma* (Ingrouille et al., 2002; Rossetto et al., 2002, 2007; Soejima and Wen, 2006; Wen et al., 2007, 2013; Ren et al., 2011; Trias-Blasi et al., 2012; Zhang et al., 2015b; Lu et al., in press). Lu et al. (2013), in particular, reconstructed the phylogeny and biogeography of *Cayratia* Juss. with 27 representatives of *Tetrastigma*, which supported that *Tetrastigma* belongs to the *Cayratia-Cyphostemma-Tetrastigma* (CCT) clade and is sister to the newly separated genus *Causonis* Raf. from *Cayratia* (Wen et al., 2013, 2015a). A comprehensive phylogenetic analysis of *Tetrastigma* was conducted by Chen et al. (2011a) based on four plastid markers (*atp*B*-rbc*L, *rps*16, *trn*L*-trn*F, and *psb*A*-trn*H) for 53 species and four varieties. The monophyly of *Tetrastigma* was confirmed and eight major clades within *Tetrastigma* were identified, although some of the major clades were not well-supported and the relationships among these clades were not resolved. Their study also provided insights into understanding the evolutionary patterns of leaf and tendril architecture. However, neither of these studies has assessed the taxonomic importance of seed morphology within a phylogenetic framework.

The aims of our study are to: (1) reconstruct a robust phylogeny of *Tetrastigma* based on an extensive taxon and character sampling; (2) document the comparative seed morphology of *Tetrastigma* and reconstruct the evolutionary history for key seed characters; and (3) detect morphological support for the major clades of *Tetrastigma* and evaluate previous infrageneric classifications within a phylogenetic framework.

## Materials and methods

### Taxon sampling, DNA extraction, polymerase chain reaction (PCR), and sequencing

Extensive field investigations have been conducted in China, Indonesia, Myanmar, Vietnam, and Australia to collect specimens and seed materials of *Tetrastigma* since 2012. A total of 72 species and two varieties of *Tetrastigma* were included from all major biogeographic regions of *Tetrastigma*. *Causonis japonica* (Thunb.) Raf. and *C. trifolia* (L.) Raf. were selected as outgroups (Lu et al., 2013).

Total genomic DNAs were extracted from silica-gel dried leaf material or sometimes from herbarium specimens using a Tiangen Biotech plant genomic DNA extraction kit (Beijing, China) following the manufacturer's protocol. Ten plastid DNA regions (*atp*B*-rbc*L, *atp*F*-atp*H, *mat*K, *psb*K*-psb*I, *rbc*L, *rpo*C1, *rps*16, *trn*C*-pet*N, *trn*H*-psb*A, *trn*L*-trn*F) were selected based on previous phylogenetic studies of Vitaceae (Soejima and Wen, 2006; Chen et al., 2011a; Lu et al., 2013, in press). PCR was carried out in 25-μL reactions containing 10–50 ng DNA, 12.5 μL 2 × Taq PCR Master Mix (Biomed, Beijing, China), and ddH_2_O with 0.4 μM forward and reverse primers, respectively. The amplification profiles of ten DNA regions followed the description in Lu et al. (in press). PCR products were examined using electrophoresis and 1.0% agarose gels. Sequencing was carried out on ABI Prism 154 Bigdye Terminator Cycle Sequencing Kit (Applied Biosystems, Foster City, CA, USA). The primers used for PCR and sequencing are listed in Supplementary Table 1. Voucher specimens and GenBank accession numbers are provided in Supplementary Table 2.

### Phylogenetic analyses

We assembled the newly generated sequences with Geneious 6.0.6 (http://www.geneious.com, Biomatters, 2013). Nucleotide sequences were aligned using MUSCLE 3.8.31 (Edgar, 2004) and then adjusted manually in Geneious.

Phylogenetic analyses were initially conducted for individual DNA regions using the maximum likelihood (ML) method in RAxML 8.2.8 (Stamatakis, 2014) on the CIPRES Science Gateway (Miller et al., 2010) under the GTR + G model with 1000 bootstrap replicates. Single gene-tree analyses did not detect well-supported topological conflicts among individual DNA regions (> 70% bootstrap support; Hillis and Bull, 1993). Thus, the ten individual plastid DNA regions were concatenated (referred to as the 10-cp data set hereafter) for further phylogenetic analyses. The partitioned ML and BI analyses were performed for the 10-cp data set and best fitting models for individual data partitions were selected by jModelTest 2 (Darriba et al., 2012) under the Akaike Information Criterion (AIC). GTR + G was found to be the most appropriate nucleotide substitution model for *atp*B*-rbc*L, *mat*K, *psb*K*-psb*I, *rps*16, *trn*C*-pet*N, *trn*H*-psb*A, and *trn*L*-trn*F; GTR + I for *atp*F*-atp*H; GTR + I + G for *rbc*L; and HKY for *rpo*C1. Bayesian analysis was performed on the CIPRES Science Gateway with MrBayes 3.2.6 (Ronquist and Huelsenbeck, 2003). Four Markov Chain Monte Carlo chains were run, each beginning with a random tree and sampling one tree every 1,000 generations of 10,000,000 generations. The standard deviation between the split frequencies established < 0.01, suggesting that a sufficient number of generations had been completed. The first 25% (2,500 trees) was discarded as burn-in and the remaining trees were used to calculate a 50% majority-rule consensus tree and posterior probabilities (*PP*).

### Seed morphology and character evolution

Seed material of *Tetrastigma* was obtained from our own collections and herbarium specimens. We studied specimens from A, BM, BRI, CANB, CNS, E, GDC, HHBG, HN, IBK, IBSC, K, KUN, L, MEL, MO, NSW, NY, P, PE, SING, TCD, and US [abbreviations following Index Herbariorum; Thiers, (continuously updated)] during herbarium visits or examining high-resolution digital specimen images. The species identifications were verified by carefully examining the herbarium specimens. Synonymy was checked by consulting the literature (Supplementary Table 3) and confirmation with our own observations.

Mature fruits were boiled in water for 2 min and soaked until the fruit tissue was loosened and could be removed by hand to free the seed(s). The seeds were transversely sectioned by hand with a razor blade to show the median cross section. Cross, dorsal and ventral views of seeds were then observed and photographed with a stereomicroscope (Nikon SMZ1000 with a Nikon DXM 1200F digital camera). For four species with limited seed materials (mainly from herbarium specimens), we did not cut seeds to observe their transverse sections (*T. quadrangulum* Gagnep. and Craib, *T. pyriforme* Gagnep., *T. ellipticum* Merr., *T. curtisii* (Ridl.) Suess.). Comparative studies of seed characters were conducted based on descriptive terminologies following Tiffney and Barghoorn (1976), Tiffney (1979), Latiff (1983), Chen and Manchester (2007), and Chen (2009). Species and vouchers for seeds observed in this study are listed in Supplementary Table 4.

We conducted ancestral state reconstruction for eight seed characters that were considered significant for traditional classifications and/or comparative morphological studies of Vitaceae (Latiff, 1983; Li and Wu, 1995; Chen, 2009). These characters were (1) seed shape, (2) seed surface rumination pattern, (3) chalaza length/width ratio, (4) chalaza position, (5) ventral infold position, (6) ventral infold divergence, (7) ventral infold depth in cross section, and (8) endosperm shape. Character states for these characters were defined as follows: Seed shape: (0) elliptic, (1) obovoid-elliptic, (2) obovoid, and (3) obtriangular; seed surface rumination pattern: (0) horizontal, (1) smooth, and (2) irregular; chalaza length/width ratio: (0) ≥ 4, and (1) <4; chalaza position: (0) from apex to the base, (1) in the middle, and (2) from apex to the middle; ventral infold position: (0) entire seed length, and (1) ½–¾ of seed length; ventral infold divergence: (0) diverged from middle or above, (1) parallel, and (2) diverged from base; ventral infold depth in cross section: (0) of seed length, (1) ≤ ¼ of seed length, and (2) ½ of seed length; endosperm shape: (0) M-shaped, (1) m-shaped, (2) T-shaped, (3) irregular shaped, and (4) 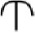-shaped (see Appendix A for details). The evolutionary history of characters 1–6 was traced over an ML tree of 46 taxa, while ancestral states for characters 7–8 were reconstructed on an ML tree of 42 taxa because transverse seed views of four taxa were unavailable. The two ML trees were reconstructed on two subsets of the 10-cp matrix by excluding taxa lacking seed information. Character evolution was reconstructed with different models in Mesquite 3.2 (Maddison and Maddison, 2015) and R 3.3.2 (R Core Team, 2016). In Mesquite, the “Trace Character History” option and the ML approach with the Markov k-state one-parameter (Mk1) model were applied (Lewis, 2001). We explored different evolutionary models for character evolution in R: the ER model (equal rates), the SYM model (symmetrical), and the ARD (all-rates-different) model as implemented in the package “ape” with the function *ace* (Paradis et al., 2004). The models with the lowest AIC score were selected to infer the ancestral states for each character.

## Results

### Phylogenetic analyses

A total of 566 new sequences were generated for *Tetrastigma* in this study. The final aligned positions of the combined 10-cp data set was 8508 base pairs (bps): *atp*B*-rbc*L with 882 bps, *atp*F*-atp*H with 770 bps, *mat*K with 1175 bps, *psb*K*-psb*I with 388 bps, *rbc*L with 1374 bps, *rpo*C1 with 804 bps, *rps*16 with 814 bps, *trn*C*-pet*N with 869 bps, *trn*H*-psb*A with 486 bps, and *trn*L-F with 946 bps, respectively.

**Figure 2:**
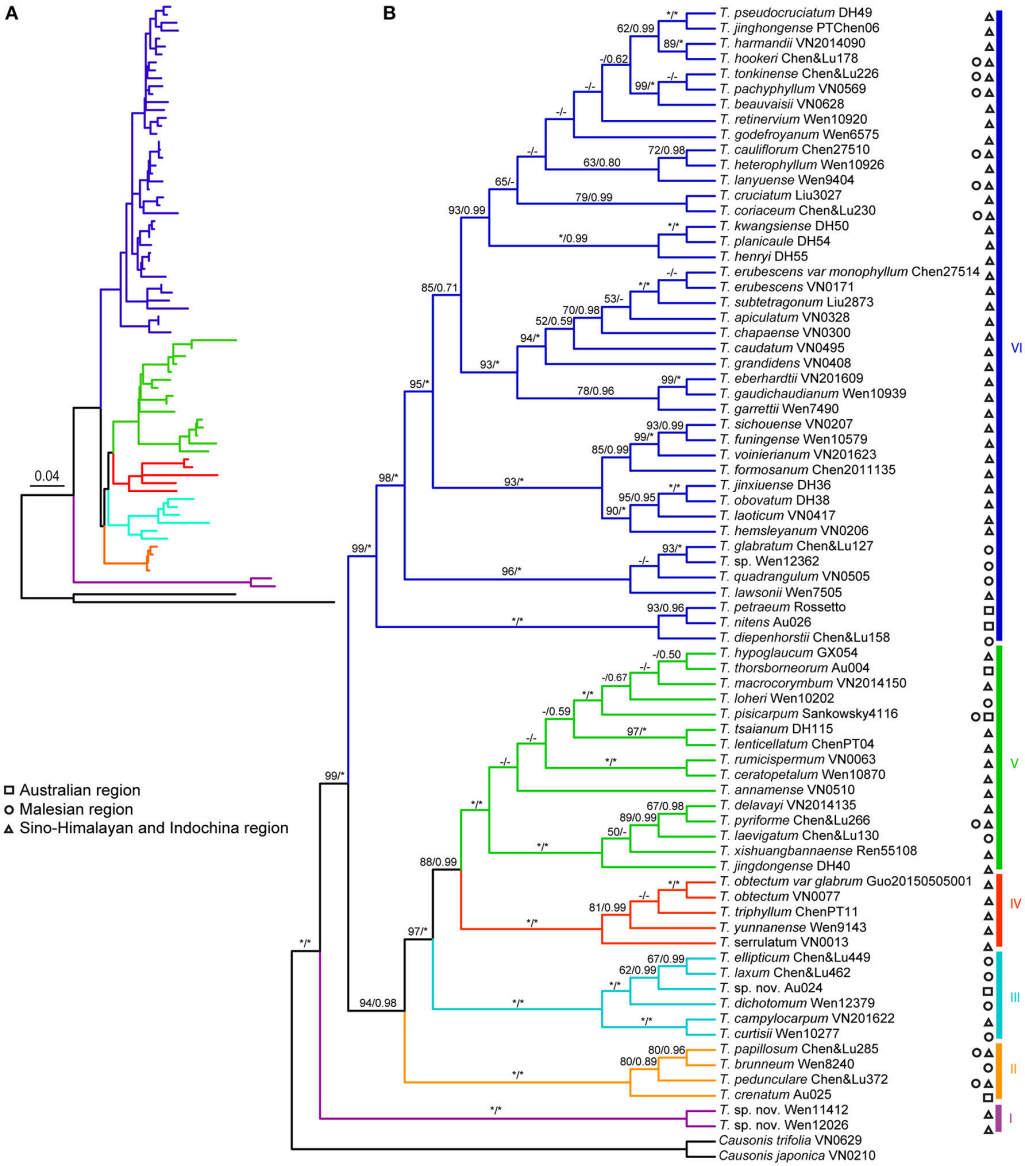
**Maximum likelihood (ML) tree for Vitaceae based on the combined 10-cp data set (*atp*B*-rbc*L, *atp*F*-atp*H, *mat*K, *psb*K*-psb*I, *rbc*L, *rpo*C1, *rps*16, *trn*C*-pet*N, *trn*H*-psb*A, *trn*L*-trn*F)**. **(A)** A phylogram overview is shown in the upper left-hand corner with the scale bar indicating the average number of nucleotide substitutions per site. **(B)** Six major clades designated in this study are highlighted with different color branches and vertical bars on the cladogram. ML bootstrap and Bayesian *PP*-values are indicated above branches (^“*”^ represents *BS* = 100% or *PP* = 1.00; “–” indicates *BS* < 50% and *PP* < 0.5). Biogeographic distribution regions of *Tetrastigma* follow Chen et al. (2011a,b) and are indicated with symbols at the right of each taxon name: the Australian region (square), the Malesian region (circle), and Sino-Himalayan and Indochina region (triangle).

The ML analyses for individual DNA regions were mostly congruent, but relationships among major clades were poorly resolved. The 10-cp data set retrieved a well-supported topology for *Tetrastigma*. The final ML optimization likelihood was -ln*L* = −22619.57. Topology of the BI analysis was mostly congruent with the ML tree. Thus, the ML tree with bootstrap (*BS*) and Bayesian posterior probability (*PP*) values is presented in Figure 2.

Our molecular analyses recognized six well-supported clades in *Tetrastigma*. Two undescribed species (*Tetrastigma* sp. nov. *Wen 12026*, and *Tetrastigma* sp. nov. *Wen 11412*) from southeastern China formed the first diverged lineage (clade I; *PP* = 1.00, *BS* = 100%; Figure 2). All the remaining taxa grouped in another well-supported lineage (*PP* = 1.00 and *BS* = 99%; Figure 2). Clades II–V formed a well-supported monophyletic group (*PP* = 0.98; *BS* = 94%), which was supported as sister to clade VI. We consider them as four separate clades (clades II, III, IV, and V) in this study due to the absence of distinct morphological characters to support this group as a single clade. The strongly supported clade VI (*PP* = 1.00; *BS* = 99%; Figure 2) includes the largest number of species of *Tetrastigma* (ca. 41 species).

### Seed morphology

**Figure 3:**
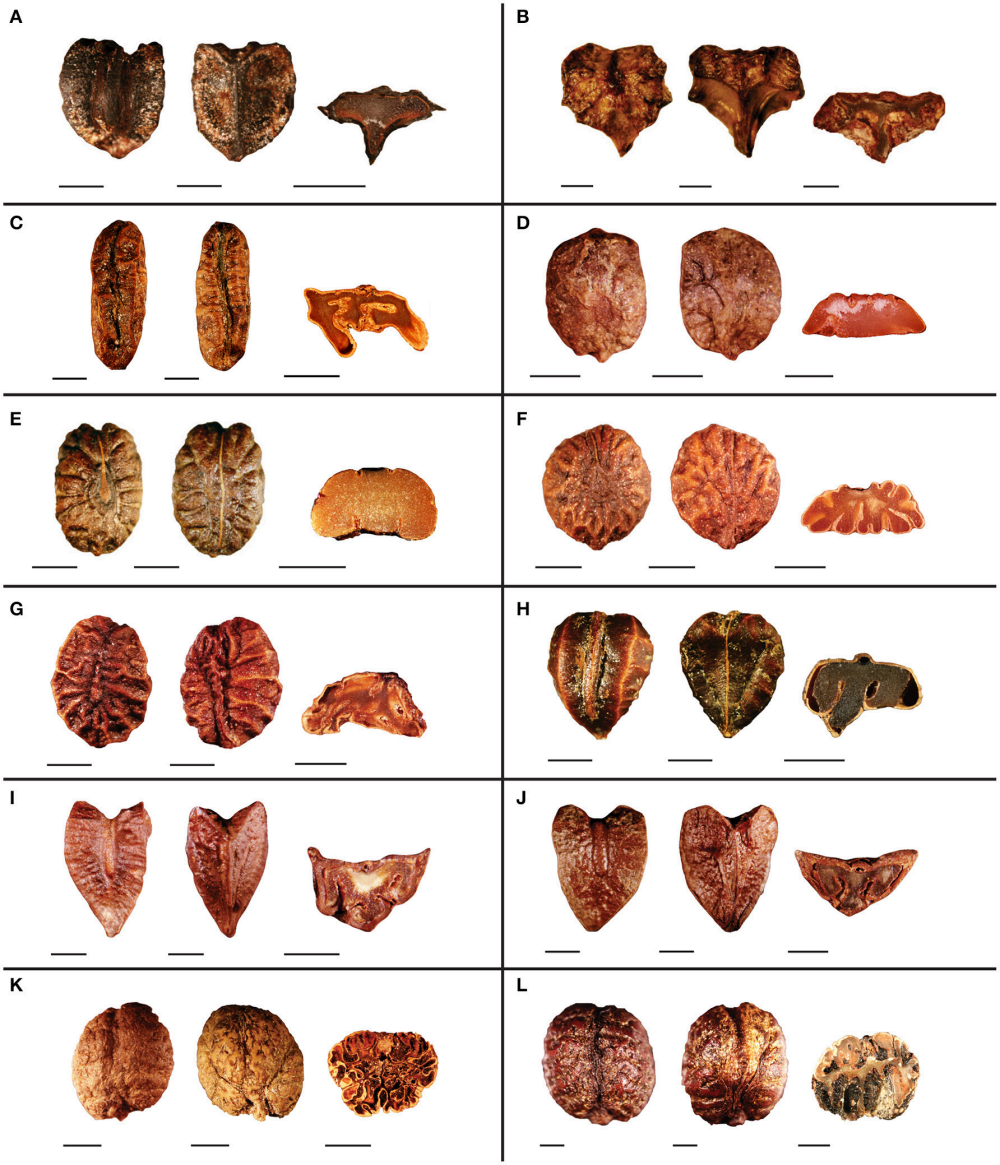
**Seed morphology of representative species from clade I–V of *Tetrastigma* (A:** clade I, **B**: clade II, **C**: clade III, **D–G**: clade IV, and **H–L**: clade V**)**. Dorsal, ventral and cross-sectional views are presented from left to right. **(A)** *Tetrastigma* sp. nov. *Wen 12026*, **(B)** *T. pedunculare* Planch., **(C)** *T. campylocarpum* Planch., **(D)** *T. yunnannese* Gagnep., **(E)** *T. obtectum* (Wall. ex M.A. Lawson) Planch. ex Franch., **(F)** *T. triphyllum* (Gagnep.) W. T. Wang, **(G)** *T. serrulatum* (Roxb.) Planch., **(H)** *T. hypoglaucum* Planch. ex Franch., **(I)** *T. delavayi* Gagnep., **(J)** *T. rumicispermum* (M.A. Lawson) Planch., **(K)** *T. thorsborneorum* Jackes, **(L)** *T. xishuangbannaense* C.L. Li. Scale bars: **(A,C–L)** = 2 mm; **(B)** = 0.5 mm.

**Figure 4:**
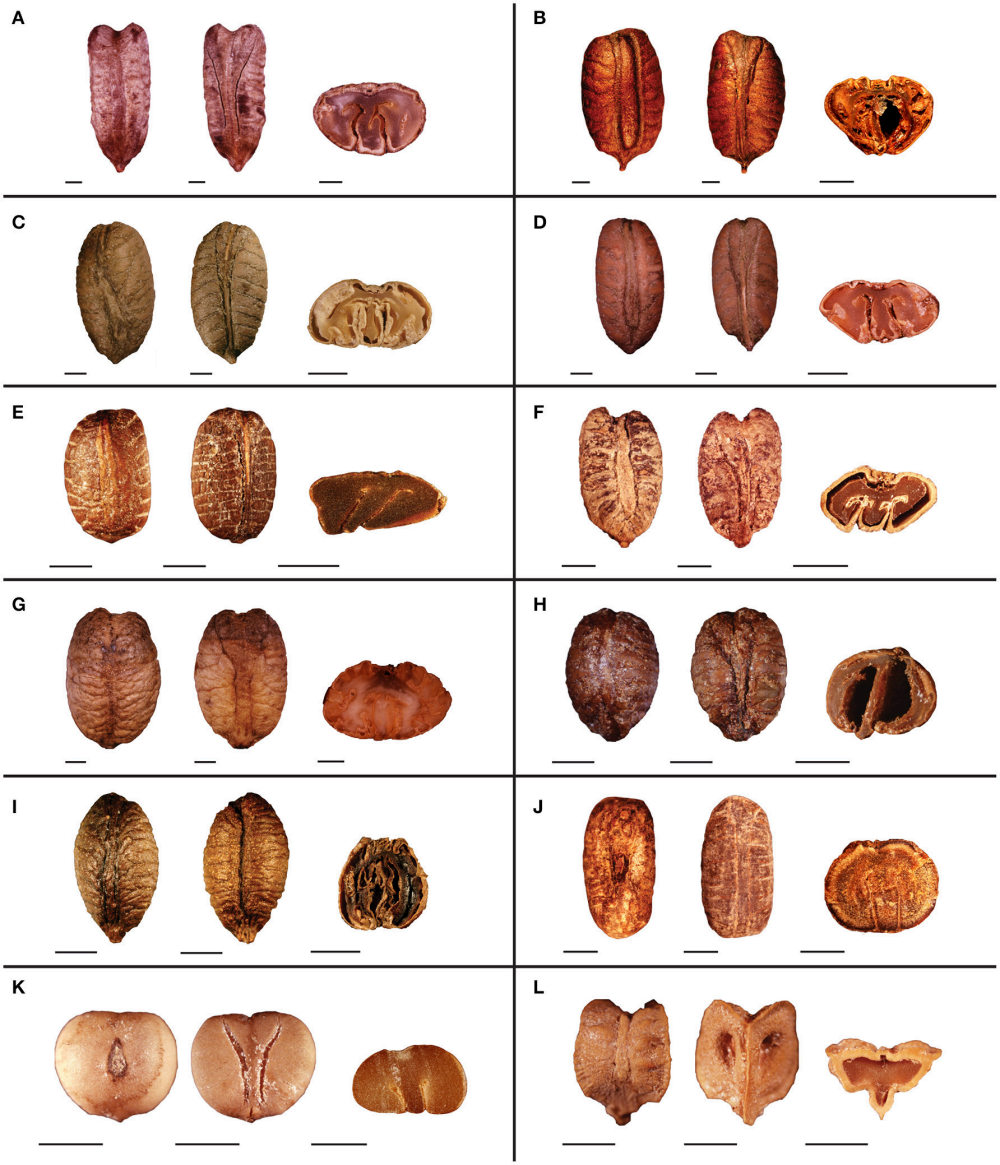
**Seed morphology of representative species from clade VI of *Tetrastigma***. Dorsal, ventral and cross-sectional views are presented from left to right. **(A)** *T. jinghongense* C.L. Li, **(B)** *T. cauliflorum* Merr., **(C)** *T. sichouense* C.L. Li, **(D)** *T. laoticum* Gagnep., **(E)** *T. henryi* Gagnep., **(F)** *T. pachyllylum* (Hemsl.) Chun, **(G)** *T. obovatum* Gagnep., **(H)** *T. retinervum* Planch., **(I)** *T. petraeum* Jackes, **(J)** *T. caudatum* Merr. and Chun, **(K)** *T. hemsleyanum* Diels and Gilg, **(L)** *T. formosanum* (Hemsl.) Gagnep. Scale bars: **(A–L)** = 2 mm.

Seed morphology of representative species from clades I–VI are presented in Figures 3A–L and Figures 4A–L. Seed size of the observed species ranges from 2 mm in *Tetrastigma pedunculare* Planch. (Figure 3B) to 18 mm in *T. jinghongense* C.L. Li (Figure 4A). Seed shape is usually obovoid-elliptic (Figures 3A,F–H, 4K,L), obtriangular (Figures 3B,I,J), elliptic (Figures 3C–E, 4A–J), or obovoid (Figures 3K,L). Sclerotesta thickness is moderate to thin with a uniseriate layer of columnar epidermal cells. The raphal ridge is less prominent on the ventral side and ruminations on the seed surface appear to be radiating away from the center toward the margins. The pattern of these ruminations is mostly horizontal or irregular, however, *T. hypoglaucum* Planch. ex Franch. (Figure 3H), *T. hemsleyanum* Diels and Gilg (Figure 4K), and *T. tonkinense* Gagnep. have smooth seeds lacking ruminations based on our observation, however, there are five ruminations on the ventral side of *T. hemsleyanum* in Figure 1–10C of Chen (2009). Seeds of most species have prominent beaks with an acute or acuminate apex but inconspicuous beaks were also found, for example in *T. caudatum* Merr. and Chun (Figure 4J). The chalaza is not sunken deeply into the seed surface and the apical notch spreads at a wider angle (≥ 155°) in most of the species with obovoid-elliptic and elliptic seeds (Figures 3B–H,K,L, 4B–E,G–K). However, the apical margin is conspicuously grooved for most species with obtriangular seeds (Figures 3I,J). An elongated chalazal knot (length/width ratio ≥ 4) is present on the dorsal side of the seed surface that extends up to the middle (Figures 3B,I,J, 4L) or the entire surface (Figures 3A,C,H,K,L, 4A–I), whereas the chalaza resides in the center of the dorsal surface for most species with an oval to pyriform chalaza (length/width ratio <4) (Figures 3D–F, 4J,K). A pair of ventral infolds present on the seed surface usually diverges away from each other toward the margins at different positions (Figures 3B–L, 4A–I,K,L), but sometimes these ventral infolds remain parallel from the base to the apex (Figures 3A, 4J). Ventral infolds diverged from the base covered ½–¾ of total seed surface. Parallel and ventral infolds diverged from the middle or above and usually cover the entire seed surface except in *T. papillosum* Planch. and *T. pedunculare* (Figure 3B) and *T. formosanum* (Hemsl.) Gagnep. (Figure 4L), which have ventral infolds that diverged above the middle but do not cover the entire seed length. Broad, circular ventral infolds are less common in *Tetrastigma* and are only observed in *Tetrastigma* sp. nov. *Wen 12026* (Figures 3A) and *T. formosanum* (Figure 4L) based on our current sampling. Endosperm shape in cross section is highly variable for the examined seeds. In addition to the common T-shaped (Figures 3A,B, 4L) and M-shaped (Figures 3C,F, 4A–K) endosperm, 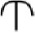-shaped (Figures 3G–J) and m-shaped (Figures 3D,E) were also observed. Moreover, rugal constrictions with ventral infolds sometimes inserted deeply into the endosperm, forming an irregular shaped endosperm (Figures 3K,L).

### Seed character evolution

**Figure 5:**
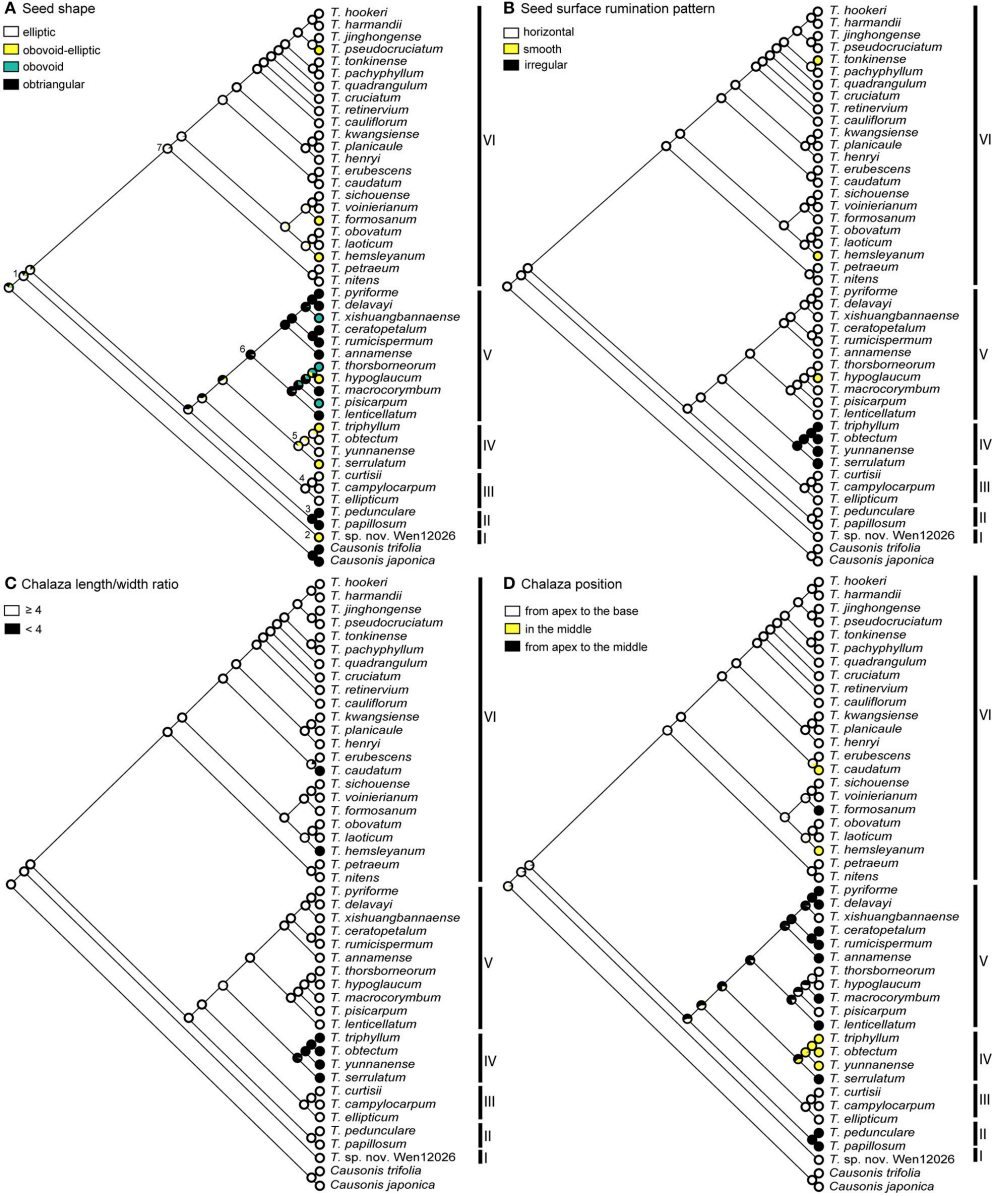
**Optimization of key seed characters (1–4) based on Mk1 model implemented in Mesquite. (A)** seed shape (character 1), **(B)** seed surface rumination pattern (character 2), **(C)** chalaza length/width ratio (character 3), and **(D)** chalaza position (character 4) inferred on a 46-terminal ML tree. Seven key nodes are marked in **(A)**.

**Figure 6:**
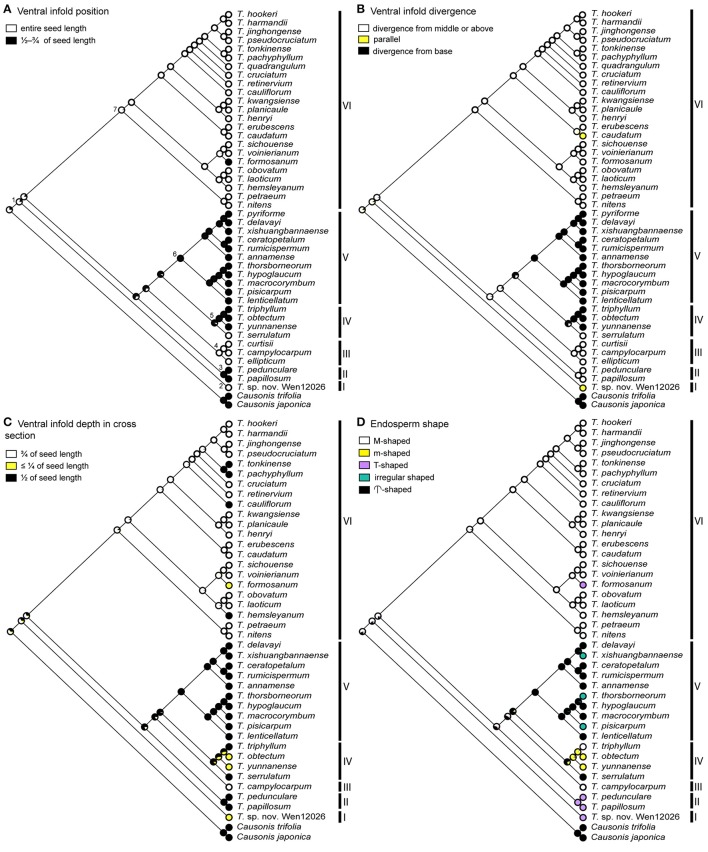
**Optimization of key seed characters (5–8) based on Mk1 model implemented in Mesquite. (A)** ventral infold position (character 5), and **(B)** ventral infold divergence (character 6) inferred on a 46-terminal ML tree. **(C)** Ventral infold depth in cross section (character 7), and **(D)** endosperm shape (character 8) inferred on a 42-terminal ML tree. Seven key nodes are marked in **(A)**.

Ancestral state reconstruction based on the Mk1 model in Mesquite is shown in Figures 5, 6. Among the three models implemented in ape, SYM was recognized as the best model for character 1 and the ER was best for characters 2, 4, and 6–8. For characters 3 and 5, both ER and SYM were suggested as best models, and ER was selected in this case to avoid over-parameterization. Ancestral state reconstruction in ape with the best models (ER or SYM) for eight characters is provided in Supplementary Figures 2, 3.

Comparison of ancestral states and likelihood proportions for key nodes of *Tetrastigma* based on the Mk1 model and the best models in R is provided in Supplementary Table 5 and congruent results were retrieved for most of the nodes (Figures 5, 6 and Supplementary Figures 2, 3).

Clades with more than one species exhibit homoplasy in most of the seed characters. Elliptic seeds are consistently present in clade III, whereas species of clades IV and VI have both obovoid-elliptic and elliptic seeds. Obtriangular seeds have evolved twice independently in clades II and V. Four species of clade V have obovoid or obovoid-elliptic seeds as well (Figure 5A and Supplementary Figure 2A). For seed surface rumination pattern, “horizontal” was inferred to be the ancestral state in *Tetrastigma* and irregular shaped surface ruminations are present only in clade IV and this character state was derived only once in *Tetrastigma* (Figure 5B and Supplementary Figure 2B). Most of the observed species in *Tetrastigma* have elongated chalazas (i.e., chalaza length/width ratio ≥ 4). Species in clade IV have oval or pyriform chalazas (chalaza length/width ratio <4). The oval chalaza has also evolved twice in clade VI (Figure 5C and Supplementary Figure 2C). The chalaza extends from the apex to the middle on the dorsal side of the seed surface in species of clade II, and extends near to the base in clades I and III, as well as in most species of clade VI. Species with a chalaza in the middle of the seed surface are confined to clades IV and VI (Figure 5D and Supplementary Figure 2D). The position of ventral infolds was nearly consistent with their divergence. Two conspicuously broad parallel ventral infolds are present on the entire seed surface in *Tetrastigma* sp. nov. *Wen 12026* (clade I). Clades II, III, and most species of clade VI have ventral infolds diverged from the middle or above (Figures 6A,B and Supplementary Figures 3A,B). Although ventral infold depth in cross section shows high variability among the species of clade IV, the species of clades II and V consistently have ventral infold depth restricted to the middle of the seed length in cross section. Ventral infolds are inserted to of seed cross section length in clade III and most species of clade VI (Figure 6C and Supplementary Figure 3C). M-shaped endosperm is present in clade III and most species of clade VI (except the T-shaped endosperm of *T. formosanum*, Figure 4L). For clades I and II, all the observed species have T-shaped endosperm. Species with m-shaped and irregular shaped endosperm are nested in clades IV and V, respectively (Figure 6D and Supplementary Figure 3D).

## Discussion

### Phylogenetic relationships

Chen et al. (2011a) recognized eight major clades within *Tetrastigma* by sampling 53 species and using four chloroplast DNA regions. Our study expanded the taxon sampling to 72 species, particularly including all five Australian species that have not been sampled by Chen et al. (2011a). Six strongly supported clades (*PP* > 0.95 and *BS* > 85%) were recognized within *Tetrastigma* on the basis of ten plastid DNA regions (Figure 2). These clades roughly correspond to the phylogeny inferred by Chen et al. (2011a) (Table 1). Clades II and IV of our study are consistent with their clades G and C, respectively; however, clade III combines taxa from clades D and E, and clade VI includes taxa from clades A, B, and H of Chen et al. (2011a). The relationships among the six major clades are strongly supported by our 10-cp data set.

**Table 1:** **A comparison of previous classifications (Latiff, 1983; Li and Wu, 1995) and phylogeny (Chen et al., 2011a) of *Tetrastigma* with major clades resolved by this study**.

**Subgenus**	**Section**	**Subsection**	**Species**	**Chen et al. (2011a)**	**This study**
*Tetrastigma*	*Tetrastigma*	*Laevia*	*T. tonkinense*	A	VI
		*Tetrastigma*	T. apiculatum, T. caudatum, T. cauliflorum, T. cruciatum, T. erubescens, T. funingense, T. godefroyanum, T. henryi, T. hookeri, T. jinghongense, T. jinxiuense^*^, T. kwangsiense^*^, T. coriaceum^*^, T. obovatum, T. pachyphyllum, T. planicaule, T. pseudocruciatum^*^, T. retinervum, T. sichouense, T. subtetragonum^*^	A	
			*T. lawsonii*	B	
			T. xishuangbannaense^*^	Not sampled	V
			T. campylocarpum, T. curtisii, T. dichotomum^*^	E	III
			T. dubium^*^, T. lincangense^*^, T. lineare^*^, T. longipedunculatum^*^, T. papillatum^*^, T. pubinerve^*^, T. scortechinii^*^, T. venulosum^*^, T. xizangense^*^, T. yiwuense^*^	Not sampled	Not sampled
	*Carinata*		T. ceratoperalum, T. delavayi, T. jingdongensis^*^, T. lenticellatum, T. macrocorymbum^*^, T. pyriforme, T. rumicispermum, T. tsaianum^*^	F	V
			*T. papillosum, T. pedunculare*	G	II
	*Orbicularia*		T. formosanum^*^, *T. hemsleyanum, T. lanyuense*	A	VI
			T. hypoglaucum^*^	Not sampled	V
			*T. serrulatum*	C	IV
*Palmicirrata*			*T. obtectum, T. triphyllum, T. yunnanense*	C	IV
Unassigned taxa			T. beauvaisii, T. chapaense^*^, T. eberhardtii, T. garrettii, T. gaudichaudianum, T. grandidense^*^, T. harmandii^*^, T. heterophyllum, T. laoticum, T. nitens^*^, T. petraeum^*^, T. quadrangulum^*^, *T. voinierianum*	A	VI
			*T. diepenhorstii*	H	
			*T. glabratum*	B	
			T. annamense, T. laevigatum, T. loheri, T. pisicarpum, T. thorsborneorum^*^	F	V
			*T. ellipticum, T. laxum*	D	III
			T. brunneum, T. crenatum^*^	G	II

* *Indicates species not sampled in Chen et al. (2011a)*.

The six major clades of our study do not completely correspond to their distributions. Species in clades I and IV are mainly from the Sino-Himalayan and Indochina region (Figure 2). However, species of clades II, III, V, and VI are distributed in all biogeographic regions of *Tetrastigma* (Figure 2). In addition, we find that most subgenera and sections in previous classifications of *Tetrastigma* are not monophyletic on our phylogenetic tree (Figure 2, Table 1).

Clade I with two undescribed species from southeastern China is sister to a clade containing all the other species of *Tetrastigma* (Figure 2). These two species seem to be closely related to *Causonis* by sharing circular ventral infolds (vs. linear ventral infolds in most species of *Tetrastigma*). However, their distinct 4-lobed stigma and the molecular data (*PP* = 1.00; *BS* = 100%) confirm their position within *Tetrastigma* (Wen et al., in preparation).

Three species from Southeast Asia (*T. brunneum* Merr., *T. papillosum*, and *T. pedunculare*) and *T. crenatum* Jackes from Australia form the strongly supported clade II (*PP* = 1.00; *BS* = 100%; Figure 2). *Tetrastigma papillosum* and *T. pedunculare* were included in section *Carinata* by Latiff (1983), but the two species can be distinguished from other species of section *Carinata* by their relatively smaller seeds (< 0.5–0.8 cm), ridged seed surface, and long and conspicuous beak based on the key provided by Latiff (1983).

Clade III has species mainly distributed in the Malesian region, but also includes *T. campylocarpum* Planch. from the Sino-Himalayan and Indochina region, and an undescribed species (*Tetrastigma* sp. nov. *Au027*) from Australia (*PP* = 1.00; *BS* = 100%; Figure 2). *Tetrastigma campylocarpum, T. dichotomum* Planch., and *T. curtisii* belong to section *Tetrastigma*, whereas the taxonomic position of *T. ellipticum*, and *T. laxum* Merr. has not been checked before (Table 1).

Species of clade IV are widely distributed in the Sino-Himalayan and Indochina region (*PP* = 1.00; *BS* = 100%; Figure 2). Clade IV includes *T. serrulatum* (Roxb.) Planch. of section *Orbicularia* and all three species of subgenus *Palmicirrata* that are distinct in having digitately branched tendrils (Table 1). Clearly, subgenus *Tetrastigma* circumscribed by Li and Wu (1995) is not monophyletic, as subgenus *Palmicirrata* is nested within the former (Figure 2). The two subgenera of *Tetrastigma* based on the branching pattern of tendrils are thus not appropriate although the digitately branched tendrils have evolved only once in *Tetrastigma* (Chen et al., 2011a).

Species of clade V (*PP* = 1.00; *BS* = 100%; Figure 2) roughly correspond to section *Carinata* (Table 1). Characteristics of section *Carinata*, such as obtriangular seeds, tuberculate seed ornamentation, chalazal extensions from the apex to the middle of the dorsal surface, and T-shaped endosperm are inconsistent in the context of our phylogenetic analyses. Species of clade V also have obovoid (Figures 3K,L) and obovoid-elliptic seeds with smooth surfaces and a fully extended chalaza (Figure 3H). Clade V contains representatives from geographically diverse regions of *Tetrastigma* including the Australian species *T. thorsborneorum* Jackes. This clade also includes species from different sections defined by Li and Wu (1995): *T*. *hypoglaucum* of section *Orbicularia* and *T. xishuangbannaense* C.L. Li of section *Tetrastigma*.

More than 50% of the sampled species (41 spp.) were included in a morphologically diverse clade VI with high support (*PP* = 1.00; *BS* = 99%; Figure 2). Most species in this clade belong to section *Tetrastigma* (Table 1). Although this clade is highly diverse in its vegetative morphology as well as fruit and seed characters, taxa of clade VI share ventral infolds diverging from the middle or above the seed surface (rarely parallel, Figure 4J). These species are widely distributed in all geographic regions of *Tetrastigma* (Figure 2). The Australian species *T. nitens* (F. Muell.) Planch. and *T. petraeum* Jackes formed the first diverged lineage of clade VI. The two species are morphologically similar with *T. petraeum* having larger, broader leaves, and less pulpy berries (Jackes, 1989). Furthermore, three species of section *Orbicularia* (i.e., *T. hemsleyanum, T. formosanum* and *T. lanyuense* C.E. Chang) were resolved to be members of clade VI.

### Evolution of seed morphology

The taxonomic and phylogenetic significance of seed morphology in several vitaceous genera has been examined in previous studies (Chen and Manchester, 2007, 2011; Popova and Yakovleva, 2009; Latiff, 2012; Lu et al., 2013). Chen (2009) provided a brief overview of *Tetrastigma* seed morphology (Supplementary Figures 1B–K). We herein systematically investigate seed morphology of *Tetrastigma* for infrageneric classification with ca. 60% of the species sampled. Furthermore, we traced the evolution of eight key characters within a well-resolved phylogenetic framework (Figures 5, 6 and Supplementary Figures 2, 3). Despite the high incidence of homoplasy observed within the main phylogenetic groups, these characters are useful to provide morphological support for the major clades of *Tetrastigma*. Here we test the taxonomic importance of these characters in the context of a robust *Tetrastigma* phylogeny (Figures 5, 6 and Supplementary Figures 2, 3).

Species with obtriangular seeds were associated with section *Carinata*, while section *Tetrastigma* contains elliptic, obovoid-elliptic or obovoid seeds (Latiff, 1983; Li and Wu, 1995; Table 1). However, character optimization suggests that “seed shape” is not an appropriate character for infrageneric classification as states of this character have evolved multiple times within *Tetrastigma* (Figure 5A and Supplementary Figure 2A).

Ornamentation of the seed surface was a taxonomically important character to identify infrageneric groups of *Tetrastigma* (Latiff, 1983; Li and Wu, 1995). However, it is difficult to delimit character states of ornamentation of seed surface (pers. observation, SH). For example, rugulose to corrugated or tuberculate to ridged seed surfaces are sometimes observed in the same species. We thus optimized “rumination pattern” instead of “ornamentation” of seed surface (Figure 5B and Supplementary Figure 2B). Rumination pattern is evolutionarily important for the taxonomy of *Tetrastigma*: “irregular” ruminations only occurred in clade IV and having “horizontal” ruminations is a shared ancestral state for all other major clades.

Chen and Manchester (2007, 2011) emphasized the importance of the length and shape of chalaza and ventral infolds for distinguishing vitaceous seeds at the generic level. According to Chen and Manchester (2007), morphologically diverse *Tetrastigma* seeds could be distinguished by a dorsal oval to elongated chalaza that is not sunken deep into the seed surface and a pair of long ventral infolds. The ventral infolds either diverge away from each other giving a V- or Y-shaped appearance or remain parallel. In our character optimization, patterns of variation for the chalaza length/width ratio remain consistent among the major clades except for taxa in the highly diverse clade VI (Figure 5C and Supplementary Figure 2C). Although the chalaza position shows a complex evolutionary pattern in clades V and VI, it is correlated with chalaza shape to distinguish other clades. For example, clades II and III have an elongated chalaza, but the position of the chalaza is variable among these clades. Chalazas usually extend from the apex to the middle (of seed length) for clade II and from the apex to the base for taxa in clade III. In addition, an oval to pyriform chalaza usually resides in the middle of the dorsal surface except in *T. serrulatum* (Figure 3G), which has a pyriform chalaza covering the upper half of the seed dorsal surface (Figure 5D and Supplementary Figure 2D).

The position and divergence of ventral infolds (Figures 6A,B and Supplementary Figures 3A,B) are partially correlated with each other. Ventral infolds diverge at variable positions on the seed surface and remain parallel in a few taxa. Ventral infolds diverge from the base in clades IV and V, never reaching the apical margin and always occupy ½–¾ of seed length. Ventral infolds that diverge from the middle or above and covering the entire seed length are found in most of the observed species of clades III and VI with a few exceptions (see Figures 3B, 4L). For Malesian species, parallel ventral infolds were associated with section *Tetrastigma* by Latiff (1983). Of the 44 *Tetrastigma* species we observed, only *T. caudatum* and *Tetrastigma* sp. nov. *Wen 12026* have parallel ventral infolds extending from the base to the apex of seed. Divergence of ventral infolds is also a useful character: *Tetrastigma papillosum* and *T. pedunculare* from clade II have ventral infolds diverged from the middle or above of seed, which is distinct from other species of section *Carinata* that have ventral infolds diverged from the base.

Endosperm shape has been used as an important character for the section-level classification of *Tetrastigma* by Latiff (1983). However, the evolutionary history of this character is very complex on our phylogenetic tree. T-shaped endosperm has evolved three times within *Tetrastigma* (clades I, II, and *T. formosanum* of VI). Most species of clades III and VI have M-shaped endosperm. However, other character states show intricate evolutionary patterns in clades IV and V (Figure 6D and Supplementary Figure 3D).

**Figure 7:**
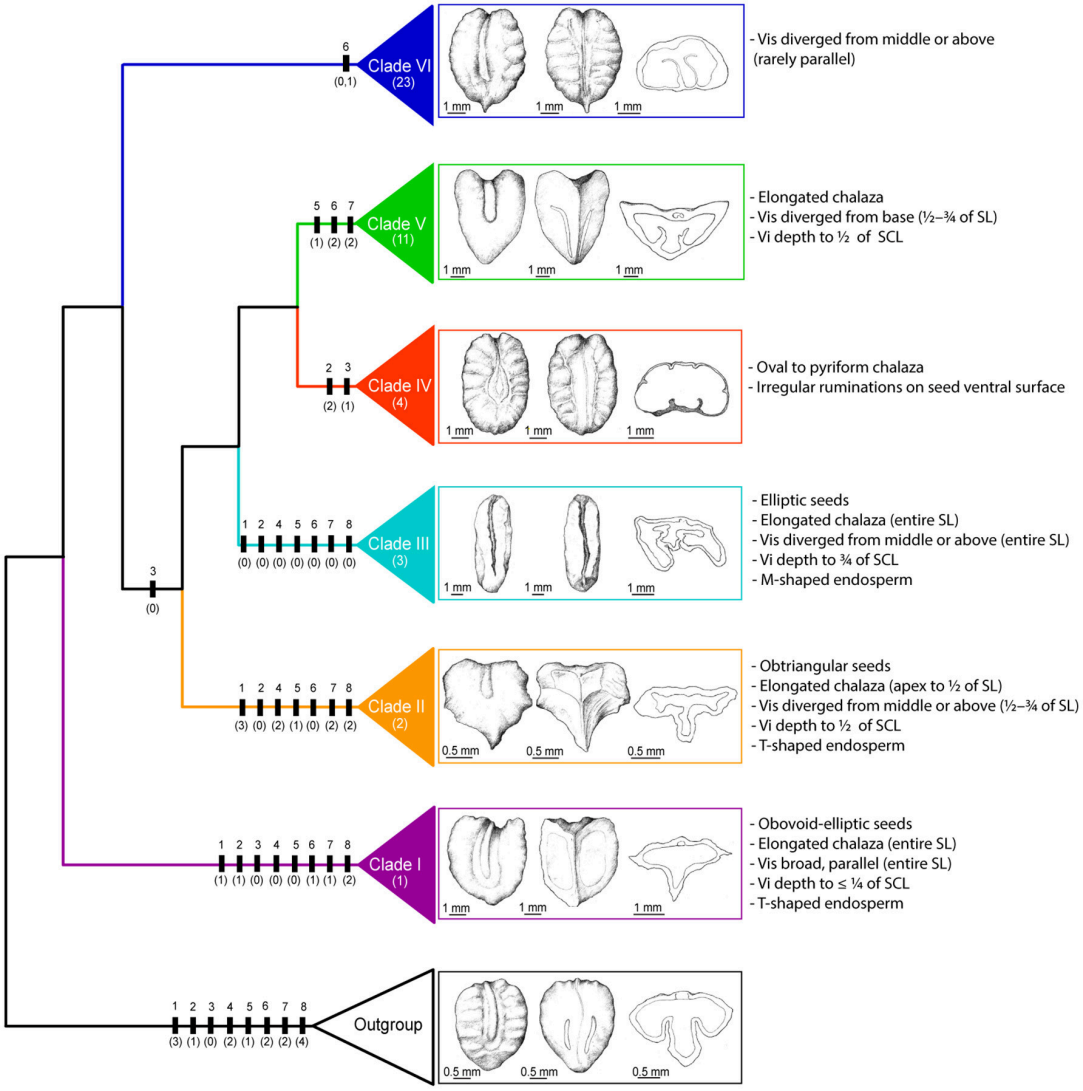
**Simplified ML cladogram showing major evolutionary lineages within *Tetrastigma*, with seed morphology indicated for representative taxa**. Outgroup, *Causonis japonica* (Thunb.) Raf.; clade I, *Tetrastigma* sp. nov. *Wen 12026*; clade II, *T*. *pedunculare* Planch.; clade III, *T*. *campylocarpum* Planch.; clade IV, *T*. *obtectum* (Wall. ex M.A. Lawson) Planch. ex Franch.; clade V, *T*. *rumicispermum* (M.A. Lawson) Planch.; clade VI, *T*. *cruciatum* W. G. Craib and Gagnep. SL, seed length; SCL, seed cross length; Vi, ventral infold; Vis, ventral infolds. Significant morphological characters are indicated on the branches using vertical bars and character number and state were shown above and below the bar, respectively. Number of taxa with seed morphology observed in each clade is indicated below the clade name. Dorsal, ventral and cross-sectional views are shown from left to right. Seed illustrations were drawn by Ai-Li Li.

Most of the seed characters optimized in our study were widely used in traditional classification systems of *Tetrastigma*. We noticed that the taxonomic groups classified by these characters are not natural because they do not reflect phylogenetic relationships among species (Figure 2, Table 1). However, it is possible to circumscribe most clades with a combination of seed morphological characters. We herein provide the diagnostic seed morphological characters for six major clades of *Tetrastigma* (Figure 7) based on 10-cp data set. Clade I is characterized by obovoid-elliptic seeds with an elongated dorsal chalaza present on the entire seed length and broad parallel ventral infolds present on the entire seed surface but inconspicuously inserted into the T-shaped endosperm. Clade II is supported by obtriangular seeds having an elongated chalaza extending from the apex to the middle of the dorsal surface and ventral infolds diverged above the middle but not covering the entire length of seed. Furthermore, species in clade II have ventral infolds moderately inserted (½ of seed cross section length) into the T-shaped endosperm. Clade III can be recognized by elliptic seeds with an elongated dorsal chalaza and ventral infolds diverged from the middle or above, both covering the entire seed surface, ventral infolds inserted deeply (of seed cross section length) into the M-shaped endosperm. Species of clade IV have seeds with irregular shaped ruminations on the seed surface and an oval to pyriform chalaza. Taxa of clade V appear to have an elongated chalaza and ventral infolds diverged from the base covering ½–¾ of total ventral seed surface and are moderately inserted into the endosperm (½ of seed cross section length). Species of clade VI share ventral infolds diverged from the middle or above rarely parallel covering the entire ventral seed surface (except *T. formosanum*).

## Conclusions

Based on extensive taxon sampling (72/95 taxa) and a large molecular data set (ten plastid DNA regions totaling 8508 bps), we retrieved a well-resolved phylogeny of *Tetrastigma*, the host plant genus of Rafflesiaceae. Within this framework, we evaluated the traditional classification. Furthermore, we documented seed morphology of 44 species and reconstructed the ancestral states for eight taxonomically important seed characters with different evolutionary models. Detailed seed morphology and diagnostic characters for six major clades lay the foundation for an infrageneric classification of *Tetrastigma* and will facilitate the recognition of *Tetrastigma* seed fossils. More molecular data, in particular from the nuclear genome (Zimmer and Wen, 2012, 2015), will be evaluated to test the congruence between the plastid and nuclear phylogenies and further explore the charismatic parasite-host plant association between Rafflesiaceae containing the largest flower of the world and *Tetrastigma* species. A comprehensive biogeographic analysis is also required to demonstrate the rapid diversification of the whole genus especially the morphologically diverse clade VI. The present phylogenetic and seed morphological work advances our understanding of *Tetrastigma* taxonomy and evolution, and the genus offers a great opportunity for integrative multi-dimensional systematic work today (Wen et al., 2015b).

## Author contributions

LL designed the study. SH, VD, and JZ performed the research and analyzed the data. SH, VD, SI, JZ, LL, JW, and ZC discussed the data and wrote the manuscript.

## Funding

This study was supported by the National Natural Science Foundation of China (NNSF 31500179 and 31590822), and Science and Technology Basic Work (2013FY112100). Field work was partially supported by the Sino-African Joint Research Center, the Chinese Academy of Sciences (SAJC201613). SH and VD were supported by CAS-TWAS President's Fellowship for International Ph.D. Students.

### Conflict of interest statement

The authors declare that the research was conducted in the absence of any commercial or financial relationships that could be construed as a potential conflict of interest.
