# Supplementary material for: Robust Phylogeny of Tetrastigma (Vitaceae) Based on Ten Plastid DNA Regions: Implications for Infrageneric Classification and Seed Character Evolution
Source: Front Plant Sci. 2017 Apr 26;8:590. doi: 10.3389/fpls.2017.00590 (PMC5405133; doi:10.3389/fpls.2017.00590)
Supplement: Supplementary file 1 [file DataSheet1.doc]

Supplementary Material

Robust phylogeny of *Tetrastigma* (Vitaceae) based on ten plastid DNA regions: Implications for infrageneric classification and seed character evolution

**Sadaf Habib^1,2,†^, Viet-Cuong Dang^1,2,3,†^, Stefanie M. Ickert-Bond^4^, Jin-Long Zhang^5^, Li-Min Lu^1,*^, Jun Wen^6^, Zhi-Duan Chen^1,3^**

^1^ State Key Laboratory of Systematic and Evolutionary Botany, Institute of Botany, Chinese Academy of Sciences, Beijing, China

^2^University of Chinese Academy of Sciences, Beijing, China

^3^ Sino-African Joint Research Center, Chinese Academy of Sciences, Wuhan, China

^4^ UA Museum of the North Herbarium and Department of Biology and Wildlife, University of Alaska Fairbanks, Fairbanks, AK, USA

^5^ Flora Conservation Department, Kadoorie Farm and Botanic Garden, Tai Po, Hong Kong SAR, China

^6^ Department of Botany, National Museum of Natural History, Smithsonian Institution, Washington, DC, USA

*Correspondence:
Li-Min Lu
[**liminlu@ibcas.ac.cn**](mailto:liminlu@ibcas.ac.cn)

^†^These authors contributed equally to this work

# Supplementary Data

## Supplementary Figures


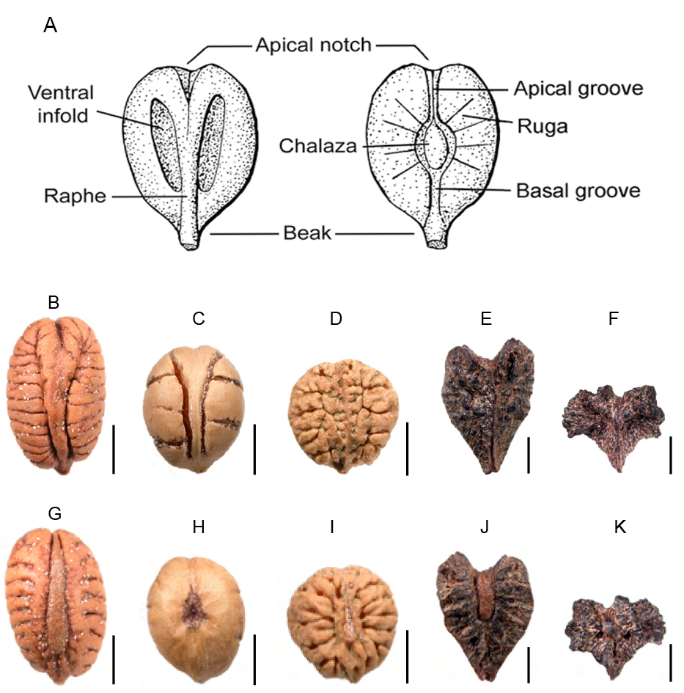


**Supplementary Figure 1. (A) Morphology and terminology of vitaceous seeds** (Adapted from Tiffney and Barghoorn, 1976 and Chen, 2009). **Morphological diversity of seeds in the genus *Tetrastigma* of Vitaceae** (Chen, 2009)**: (B)** An elliptic seed with horizontal ruminations and ventral infolds diverged from middle or above. **(C)** An obovoid-elliptic seed with smooth surface. **(D)** An obovoid seed with irregular surface ruminations. **(E)** An obtriangular seed with ventral infolds diverged from base. **(F)** An obtriangular seed with ventral infolds diverged from middle. **(G)** An elongated chalaza covering entire seed dorsal surface. **(H–I)** An oval chalaza present in the middle. **(J–K)** An elongated chalaza positioned from apex to the middle. Scale bar: = 2mm.


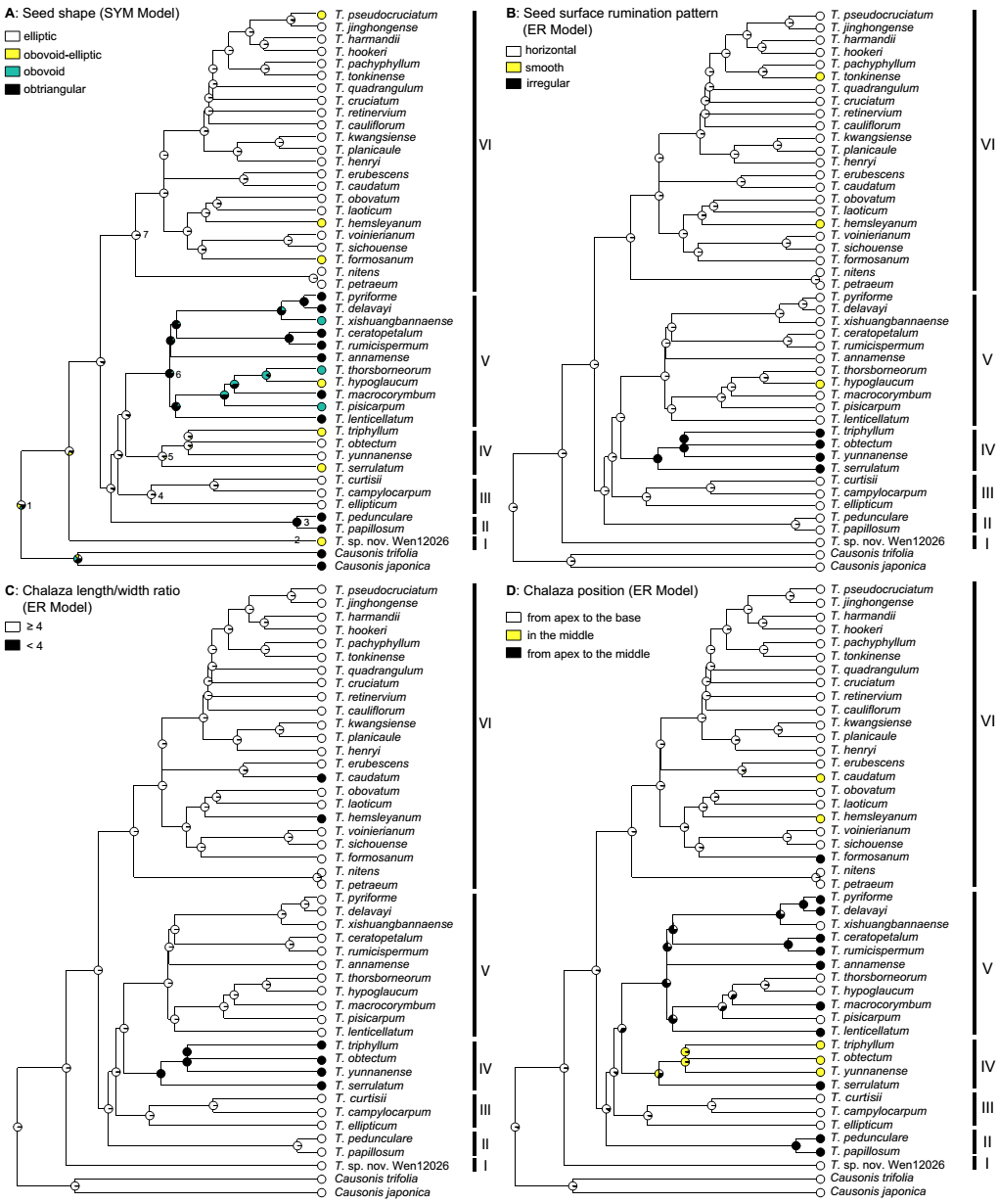


**Supplementary Figure 2. Optimization of key seed characters (1**–**4) based on the ER/SYM model implemented in R. (A)** seed shape (character 1), **(B)** seed surface rumination pattern (character 2), **(C)** chalaza length/width ratio (character 3), and **(D)** chalaza position (character 4) inferred on a 46-terminal maximum likelihood (ML) tree. Seven key nodes are marked in **(A)**.

##
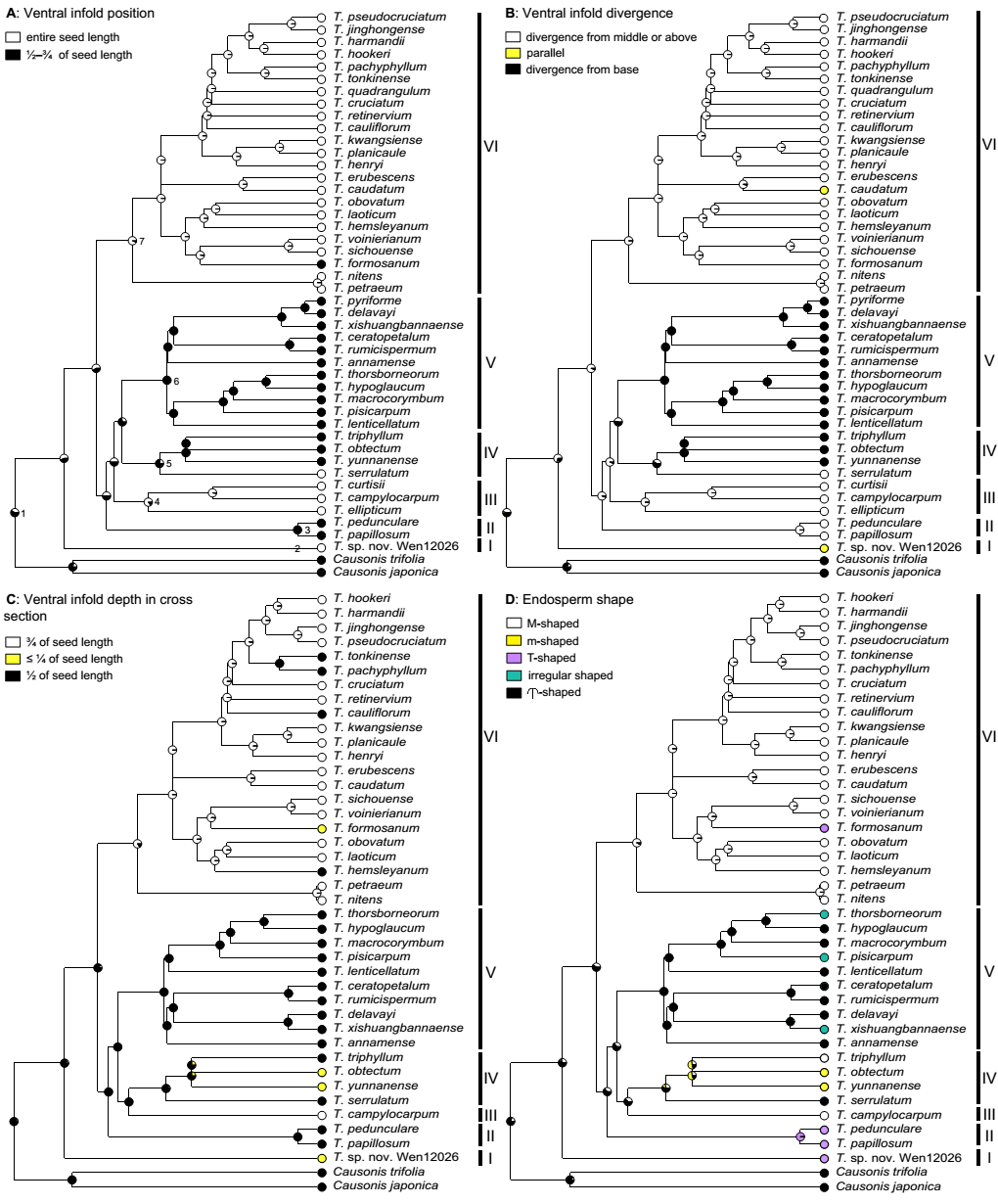


## Supplementary Figure 3. Optimization of key seed characters (5–8) based on the ER model implemented in R. (A) ventral infold position (character 5), and (B) ventral infold divergence (character 6) inferred on a 46-terminal ML tree. (C) Ventral infold depth in cross section (character 7), and (D) endosperm shape (character 8) inferred on a 42-terminal ML tree. Seven key nodes are marked in (A).

## Supplementary Tables

Supplementary Table 1. Primers used in this study for PCR and sequencing.

| **Gene Regions** | **Primer** | **Primer Sequence** | **References** |
| --- | --- | --- | --- |
| *atp*B*-rbc*L | 2F | GAAGTCGTAGGATTGATTCTC | Manen et al., 1994 |
|  | 5R | TACAGTTGTCCATGTACCAG | Manen et al., 1994 |
| *atp*F*-atp*H | atpF | ACTCGCACACACTCCCTTTCC | Fazekas et al., 2008 |
|  | atpH | GCTTTTATGGAAGCTTTAACAAT | Fazekas et al., 2008 |
| *mat*K | AF | CTATATCCACTTATCTTTCAGGAGT | Ooi et al., 1995 |
|  | 8R | AAAGTTCTAGCACAAGAAAGTCGA | Ooi et al., 1995 |
|  | Kim_F | CGTACAGTACTTTTGTGTTTACGAG | Fazekas et al., 2008 |
|  | Kim_R | ACCCAGTCCATCTGGAAATCTTGGTTC | Fazekas et al., 2008 |
| *psb*K*-psb*I | psbK | TTAGCCTTTGTTTGGCAAG | Fazekas et al., 2008 |
|  | psbI | AGAGTTTGAGAGTAAGCAT | Fazekas et al., 2008 |
| *rbc*L | 1F | ATGTCACCACAAACAGAAAC | Olmstead et al., 1992 |
|  | 1460R | TCCTTTTAGTAAAAGATTGGGCCGAG | Olmstead et al., 1992 |
| *rpo*C1 | LP1F | TATGAAACCAGAATGGATGG | Hollingsworth et al., 2009 |
|  | LP5R | CAAGAAGCATATCTTGASTYGG | Hollingsworth et al., 2009 |
| *rps*16 | F | GTGGTAGAAAGCAACGTGCGACTT | Oxelman et al., 1997 |
|  | R2 | TCGGGATCGAACATCAATTGCAAC | Oxelman et al., 1997 |
|  | V1F | AAGTGTATCGTGCGGGAATC | Chen et al., 2011 |
| *trn*C*-pet*N | F | CCAGTTCAAATCTGGGTGTC | Lee and Wen, 2004 |
|  | 2r | CCATTAAAGCAGCCCAAGCAAGAC | Lee and Wen, 2004 |
| *trn*H*-psb*A | R | CGCGCATGGTGGATTCACAAATC | Sang et al., 1997 |
|  | F | GTTATGCATGAACGTAATGCTC | Sang et al., 1997 |
| *trn*L*-trn*F | C | CGAAATCGGTAGACGCTACG | Taberlet et al., 1991 |
|  | D | GGGGATAGAGGGACTTGAAC | Taberlet et al., 1991 |
|  | E | GGTTCAAGTCCCTCTATCCC | Taberlet et al., 1991 |
|  | F | ATTTGAACTGGTGACACGAG | Taberlet et al., 1991 |
|  | f’ | ATTTTCAGTCCTCTGCTCTACC | Soejima and Wen, 2006 |

**Supplementary Table 2**. Taxon sampling and GenBank accession numbers of DNA sequences.

| **Species** | **Voucher specimen** | **Locality** | ***atp*B*-rbc*L** | ***rps*16** | ***trn*C*-pet*N** | ***trn*H*-psb*A** | ***trn*L*-trn*F** | ***atp*H*-atp*F** | ***mat*K** | ***psb*K*-psb*I** | ***rbc*L** | ***rpo*C1** |
| --- | --- | --- | --- | --- | --- | --- | --- | --- | --- | --- | --- | --- |
| *Causonis japonica* (Thunb.) Raf. | *Chen et al VN0210* (PE) | Vietnam, Tam Dao | KY766309 | KY766647 | KY766705 | KY766761 | KY766819 | KY766364 | KY766421 | KY766475 | KY766534 | KY766590 |
| *Causonis trifolia* (L.) Raf. | *Chen et al VN0629* (PE) | Vietnam, Ba Den | KY766310 | KY766648 | KY766706 | KY766762 | KY766820 | KY766365 | KY766440 | KY766476 | KY766535 | KY766591 |
| *Tetrastigma annamense* Gagnep. | *Chen et al VN0510* (PE) | Vietnam, Bi Doup | KY766311 | KY766649 | KY766707 | KY766763 | KY766821 | KY766366 | KY766441 | KY766477 | KY766536 | KY766592 |
| *Tetrastigma apiculatum* Gagnep. | *Chen et al VN0328* (PE) | Vietnam, Ba Vi | KY766312 | KY766650 | KY766708 | KY766764 | KY766822 | KY766367 | KY766442 | KY766478 | KY766537 | KY766593 |
| *Tetrastigma beauvaisii* Gagnep. | *Chen et al VN0628* (PE) | Vietnam, Ba Den | KY766313 | KY766651 | KY766709 | KY766765 | KY766823 | KY766368 | KY766443 | KY766479 | KY766538 | KY766594 |
| *Tetrastigma brunneum* Merr. | *Wen 8240* (US) | Philippines, Luzon | HM585549 | HM585825 | – | HM585689 | HM585965 | – | – | – | – | – |
| *Tetrastigma campylocarpum* Planch. | *Chen et al VN201622B* (PE) | Vietnam, Son La | KY766314 | KY766652 | KY766710 | KY766766 | KY766824 | KY766369 | KY766444 | KY766480 | KY766539 | KY766595 |
| *Tetrastigma caudatum* Merr. & Chun | *Chen et al VN0495* (PE) | Vietnam, Hon Ba | KY766315 | KY766653 | KY766711 | KY766767 | KY766825 | KY766370 | KY766445 | KY766481 | KY766540 | KY766596 |
| *Tetrastigma cauliflorum* Merr. | *Chen et al 27510* (PE) | Yunnan, China | KY766316 | KY766654 | KY766712 | KY766768 | KY766826 | KY766371 | KY766446 | KY766482 | KY766541 | KY766597 |
| *Tetrastigma ceratopetalum* C.Y. Wu | *Wen 10870* (US) | China, Yunnan | HM585558 | HM585834 | – | HM585698 | HM585974 | – | – | – | – | – |
| *Tetrastigma chapaense* Merr. | *Chen et al VN0300* (PE) | Vietnam, Tam Dao | KY766317 | KY766655 | KY766713 | KY766769 | KY766827 | KY766372 | KY766447 | KY766483 | KY766542 | KY766598 |
| *Tetrastigma coriaceum* (DC.) Gagnep. | *Chen & Lu 230* (PE) | Indonesia, Mangrove forest, Bali | KY766318 | KY766656 | KY766714 | KY766770 | KY766828 | KY766373 | KY766448 | KY766484 | KY766543 | KY766599 |
| *Tetrastigma crenatum* Jackes | *Lu & Jackes Au025* (PE) | Australia, Cairns | KY766319 | KY766657 | KY766715 | KY766771 | KY766829 | KY766374 | KY766422 | KY766485 | KY766544 | KY766600 |
| *Tetrastigma cruciatum* W.G. Craib & Gagnep. | *Liu 3027* (PE) | China, Yunnan | KY766320 | KY766658 | KY766716 | KY766772 | KY766830 | KY766375 | KY766449 | KY766486 | KY766545 | KY766601 |
| *Tetrastigma curtisii* (Ridl.) Suess. | *Wen 10277* (US) | Indonesia, SE Sulawesi | HM585563 | HM585839 | – | HM585703 | HM585979 | – | – | – | – | – |
| *Tetrastigma delavayi* Gagnep. | *Chen et al VN2014135* (PE) | Vietnam, Lao Cai | KY766321 | KY766659 | KY766717 | KY766773 | KY766831 | KY766376 | – | KY766487 | KY766546 | KY766602 |
| *Tetrastigma dichotomum* Planch. | *Wen 12379* (US) | Indonesia | KY766322 | KY766660 | KY766718 | KY766774 | KY766832 | KY766377 | KY766450 | KY766488 | KY766547 | KY766603 |
| *Tetrastigma diepenhorstii* (Miq.) Latiff | *Chen & Lu 158* (PE) | Indonesia, Bogor Botanical Garden | KY766323 | KY766661 | KY766719 | KY766775 | KY766833 | KY766378 | KY766423 | KY766489 | KY766548 | KY766604 |
| *Tetrastigma eberhardtii* Gagnep. | *Chen et al VN201609* (PE) | Vietnam, Tuyen Quang | KY766324 | KY766662 | KY766720 | KY766776 | KY766834 | KY766379 | KY766451 | KY766490 | KY766549 | KY766605 |
| *Tetrastigma ellipticum* Merr. | *Chen & Lu 449* (PE) | Indonesia, Rantepao "Kare forest" | – | KY766663 | – | KY766777 | KY766835 | KY766380 | KY766424 | KY766491 | KY766550 | KY766606 |
| *Tetrastigma erubescens* Planch. | *Chen et al VN0171* (PE) | Vietnam, Lao Cai | KY766325 | KY766664 | KY766721 | KY766778 | KY766836 | KY766381 | KY766452 | KY766492 | KY766551 | KY766607 |
| *Tetrastigma erubescens* var. *monophyllum* Gagnep. | *Chen et al 27514* (PE) | China, Yunnan | KY766326 | KY766665 | KY766722 | KY766779 | KY766837 | KY766382 | KY766453 | KY766493 | KY766552 | KY766608 |
| *Tetrastigma formosanum* (Hemsl.) Gagnep. | *Chen 2011135* (PE) | China, Taiwan | KY766327 | KY766666 | KY766723 | KY766780 | KY766838 | KY766383 | KY766454 | KY766494 | KY766553 | KY766609 |
| *Tetrastigma funingense* C.L. Li | *Wen 10579* (US) | China, Yunnan | HM585574 | HM585850 | KX925961 | HM585714 | HM585990 | – | – | – | – | – |
| *Tetrastigma garrettii* Gagnep. | *Wen 7490* (US) | Thailand, Chiang Mai | HM585578 | HM585854 | – | HM585718 | HM585994 | – | – | – | – | – |
| *Tetrastigma gaudichaudianum* Planch. | *Wen 10939* (US) | Vietnam, Hoa Binh | HM585583 | HM585859 | KX925962 | HM585721 | HM585999 | – | – | – | – | – |
| *Tetrastigma glabratum* Planch. | *Chen & Lu 127* (PE) | Indonesia, Cibodas Botanical Garden | KY766328 | KY766667 | KY766724 | KY766781 | KY766839 | KY766384 | KY766455 | KY766495 | KY766554 | KY766610 |
| *Tetrastigma godefroyanum* Planch. | *Wen 6575* (US) | China, Hainan | HM585581 | HM585857 | – | HM585719 | HM585997 | – | – | – | – | – |
| *Tetrastigma grandidens* Gagnep. | *Chen et al VN0408* (PE) | Vietnam, Ba Na | KY766329 | KY766668 | KY766725 | KY766782 | KY766840 | KY766385 | KY766456 | KY766496 | KY766555 | KY766611 |
| *Tetrastigma harmandii* Planch*.* | *Chen et al VN201490* (PE) | Vietnam, Cat Tien | KY766330 | KY766669 | KY766726 | KY766783 | KY766841 | KY766386 | KY766425 | KY766497 | KY766556 | KY766612 |
| *Tetrastigma hemsleyanum* Diels & Gilg | *Chen et al VN0206* (PE) | Vietnam, Tam Dao | KY766331 | KY766670 | KY766727 | KY766784 | KY766842 | KY766387 | KY766426 | KY766498 | KY766557 | KY766613 |
| *Tetrastigma henryi* Gagnep. | *Chen et al DH55* (PE) | China, Yunnan | KY766332 | KY766671 | KY766728 | KY766785 | KY766843 | KY766388 | KY766427 | KY766499 | KY766558 | KY766614 |
| *Tetrastigma heterophyllum* Gagnep. | *Wen 10926* (US) | Vietnam, Ninh Binh | HM585588 | HM585864 | KX925964 | HM585726 | HM586004 | – | – | – | – | – |
| *Tetrastigma hookeri* (M.A. Lawson) Planch. | *Chen & Lu 178* (PE) | Indonesia , Bogor Botanical Garden | KY766333 | KY766672 | KY766729 | KY766786 | KY766844 | KY766389 | KY766457 | KY766500 | KY766559 | KY766615 |
| *Tetrastigma hypoglaucum* Planch. ex Franch. | *H. Ren et al GX054* (PE) | China, Guangxi | – | – | KY766730 | KY766787 | – | – | – | KY766501 | – | – |
| *Tetrastigma jingdongense* C.L. Li | *Chen et al DH40* (PE) | China, Yunnan | KY766334 | KY766673 | KY766731 | KY766788 | KY766845 | KY766390 | KY766428 | KY766502 | KY766560 | KY766616 |
| *Tetrastigma jinghongense* C.L. Li | *Chen PT06* (PE) | Myanmar, Kachin | KY766335 | KY766674 | KY766732 | KY766789 | KY766846 | KY766391 | KY766458 | KY766503 | KY766561 | KY766617 |
| *Tetrastigma jinxiuense* C.L. Li | *Chen et al DH36* (PE) | China, Yunnan | KY766336 | KY766675 | KY766733 | KY766790 | KY766847 | KY766392 | KY766429 | KY766504 | KY766562 | KY766618 |
| *Tetrastigma kwangsiense* C.L. Li | *Chen et al DH50* (PE) | China, Yunnan | KY766337 | KY766676 | KY766734 | KY766791 | KY766848 | KY766393 | KY766459 | KY766505 | KY766563 | KY766619 |
| *Tetrastigma laevigatum* (Blume) Gagnep. | *Chen & Lu 130* (PE) | Indonesia, Cibodas Botanical Garden | KY766338 | KY766677 | KY766735 | KY766792 | KY766849 | KY766394 | KY766460 | KY766506 | KY766564 | KY766620 |
| *Tetrastigma lanyuense* C.E. Chang | *Wen 9404* (US) | China, Taiwan | HM585593 | HM585869 | JF437257 | HM585731 | HM586009 | – | – | – | – | – |
| *Tetrastigma laoticum* Gagnep. | *Chen et al VN0417* (PE) | Vietnam, Ba Na | KY766339 | KY766678 | KY766736 | KY766793 | KY766850 | KY766395 | KY766461 | KY766507 | KY766565 | KY766621 |
| *Tetrastigma lawsoni* (King) Burkill | *Wen 7505* (US) | Singapore, Bukit Timah Nature Reserve | HM585599 | HM585874 | – | HM585737 | HM586015 | – | – | – | – | – |
| *Tetrastigma laxum* Merr. | *Chen & Lu 462* (PE) | Indonesia, Rantepao ‘Kare forest’ | KC428768 | KY766679 | KC428795 | KC428814 | KY766851 | KY766396 | KY766430 | KY766508 | KY766566 | KY766622 |
| *Tetrastigma lenticellatum* C.Y. Wu | *Chen PT04* (PE) | Myanmar, Kachin | KY766340 | KY766680 | KY766737 | KY766794 | KY766852 | KY766397 | KY766462 | KY766509 | KY766567 | KY766623 |
| *Tetrastigma loheri* Gagnep. | *Wen 10202* (US) | Indonesia, SE Sulawesi | HM585605 | HM585880 | – | HM585743 | HM586021 | – | – | – | – | – |
| *Tetrastigma macrocorymbum* Gagnep. ex J. Wen, Boggan & Turland | *Chen et al VN2014150* (PE) | Vietnam, Kon Ka King | KY766341 | KY766681 | KY766738 | KY766795 | KY766853 | KY766398 | – | KY766510 | KY766568 | KY766624 |
| *Tetrastigma nitens* (F. Muell.) Planch. | *Lu & Jackes Au026* (PE) | Australia, Cairns | KY766342 | KY766682 | KY766739 | KY766796 | KY766854 | KY766399 | KY766431 | KY766511 | KY766569 | KY766625 |
| *Tetrastigma obovatum* Gagnep. | *Chen et al DH38* (PE) | China, Yunnan | KY766343 | KY766683 | KY766740 | KY766797 | KY766855 | KY766400 | KY766463 | KY766512 | KY766570 | KY766626 |
| *Tetrastigma obtectum* (Wall. ex M.A. Lawson) Planch. ex Franch. | *Chen et al VN0077* (PE) | Vietnam, Sa Pa | KY766344 | KY766684 | KY766741 | KY766798 | KY766856 | KY766401 | KY766464 | KY766513 | KY766571 | KY766627 |
| *Tetrastigma obtectum* var*. glabrum* (H. Lév.) Gagnep. | *Guo 20150505001* (PE) | China, Guangxi | KY766345 | KY766685 | KY766742 | KY766799 | KY766857 | KY766402 | KY766439 | KY766514 | KY766572 | KY766628 |
| *Tetrastigma pachyphyllum* (Hemsl.) Chun | *Chen et al VN0569* (PE) | Vietnam, Ca Na | KY766346 | KY766686 | KY766743 | KY766800 | KY766858 | KY766403 | KY766465 | KY766515 | KY766573 | KY766629 |
| *Tetrastigma papillosum* Planch. | *Chen & Lu 285* (PE) | Indonesia, Eka karya Botanic Garden, Bedugal | KY766347 | KY766687 | KY766744 | KY766801 | KY766859 | KY766404 | KY766432 | KY766516 | KY766574 | KY766630 |
| *Tetrastigma pedunculare* Planch. | *Chen & Lu 372* (PE) | Indonesia, East Kalimantan | KY766348 | KY766688 | KY766745 | KY766802 | KY766860 | KY766405 | KY766433 | KY766517 | KY766575 | KY766631 |
| *Tetrastigma petraeum* Jackes | *Rossetto et al., 2007* | Australia | – | – | – | – | EF179094 | – | – | – | – | – |
| *Tetrastigma pisicarpum* (Miq.) Planch. | *G. Sankowsky 4116* (CNS) | Australia, Queensland | KY766349 | KY766689 | KY766746 | KY766803 | – | KY766406 | – | KY766518 | – | KY766632 |
| *Tetrastigma planicaule* (Hook. f.) Gagnep. | *Chen et al DH54* (PE) | China, Yunnan | KY766350 | KY766690 | KY766747 | KY766804 | KY766861 | KY766407 | KY766434 | KY766519 | KY766576 | KY766633 |
| *Tetrastigma pseudocruciatum* C.L. Li. | *Chen et al DH49* (PE) | China, Yunnan | KY766351 | KY766691 | KY766748 | KY766805 | KY766862 | KY766408 | KY766466 | KY766520 | KY766577 | KY766634 |
| *Tetrastigma pyriforme* Gagnep. | *Chen & Lu 266* (PE) | Indonesia, Eka karya Botanic Garden, Bedugal | KY766352 | KY766692 | KY766749 | KY766806 | KY766863 | KY766409 | KY766435 | KY766521 | KY766578 | KY766635 |
| *Tetrastigma quadrangulum* Gagnep. & Craib | *Chen et al VN0505* (PE) | Vietnam, Hon Ba | – | KY766693 | – | KY766807 | – | – | – | KY766522 | – | – |
| *Tetrastigma retinervium* Planch. | *Wen 10920* (US) | Vietnam, Vinh Phuc | HM585625 | HM585899 | KX925969 | HM585762 | HM586040 | – | – | – | – | – |
| *Tetrastigma rumicispermum* (M.A. Lawson) Planch. | *Chen et al VN0063* (PE) | Vietnam, Sa Pa | KY766353 | KY766694 | KY766750 | KY766808 | KY766864 | KY766410 | KY766467 | KY766523 | KY766579 | KY766636 |
| *Tetrastigma serrulatum* (Roxb.) Planch. | *Chen et al VN0013* (PE) | Vietnam, Sa Pa | KY766354 | KY766695 | KY766751 | KY766809 | KY766865 | KY766411 | KY766468 | KY766524 | KY766580 | KY766637 |
| *Tetrastigma sichouense* C.L. Li | *Chen et al VN0207* (PE) | Vietnam, Tam Dao | KY766355 | KY766696 | KY766752 | KY766810 | KY766866 | KY766412 | KY766469 | KY766525 | KY766581 | KY766638 |
| *Tetrastigma* sp. | *Wen 12362* (US) | Indonesia | KY766357 | KY766698 | KY766754 | KY766811 | KY766868 | KY766414 | KY766437 | KY766527 | KY766583 | KY766640 |
| *Tetrastigma* sp. nov. | *Lu & Jackes Au024* (PE) | Australia, Cairns | KY766356 | KY766697 | KY766753 | KY766812 | KY766867 | KY766413 | KY766436 | KY766526 | KY766582 | KY766639 |
| *Tetrastigma* sp. nov. | *Wen 12026* (US) | China, Fujian | KC166377 | KC166464 | – | KC166616 | KC166682 | – | – | – | – | – |
| *Tetrastigma* sp. nov. | *Wen 11412* (US) | China, Guangdong | KC166376 | KC166463 | KC166541 | KC166615 | KC166681 | – | – | – | – | – |
| *Tetrastigma subtetragonum* C.L. Li | *Liu 2873* (PE) | China, Yunnan | KY766358 | KY766699 | KY766755 | KY766813 | KY766869 | KY766415 | KY766470 | KY766528 | KY766584 | KY766641 |
| *Tetrastigma thorsborneorum* Jackes | *Lu & Jackes Au004* (PE) | Australia, Cairns | KY766359 | KY766700 | KY766756 | KY766814 | KY766870 | KY766416 | KY766471 | KY766529 | KY766585 | KY766642 |
| *Tetrastigma tonkinense* Gagnep*.* | *Chen & Lu 226* (PE) | Indonesia, Mangrove forest, Bali | KY766360 | KY766701 | KY766757 | KY766815 | KY766871 | KY766417 | KY766472 | KY766530 | KY766586 | KY766643 |
| *Tetrastigma triphyllum* (Gagnep.) W.T. Wang | *Chen PT11* (PE) | Myanmar, Putao | KY766361 | KY766702 | KY766758 | KY766816 | KY766872 | KY766418 | KY766473 | KY766531 | KY766587 | KY766644 |
| *Tetrastigma tsaianum* C.Y. Wu | *Chen et al DH115* (PE) | China, Yunnan | KY766362 | KY766703 | KY766759 | KY766817 | KY766873 | KY766419 | KY766474 | KY766532 | KY766588 | KY766645 |
| *Tetrastigma voinierianum* Pierre ex Pit. | *Chen et al VN201623* (PE) | Vietnam, Tuyen Quang | KY766363 | KY766704 | KY766760 | KY766818 | KY766874 | KY766420 | KY766438 | KY766533 | KY766589 | KY766646 |
| *Tetrastigma xishuangbannaense* C.L. Li | *Ren 55108* (US) | China, Yunnan | – | – | JF437265 | JF437153 | KX951242 | – | – | – | – | – |
| *Tetrastigma yunnanense* Gagnep. | *Wen 9143* (US) | China, Yunnan | – | – | JF437267 | JF437155 | JF437350 | – | – | – | – | – |

*"–" indicates missing data. Abbreviations of herbaria are as follows: CNS, Australian Tropical Herbarium, Queensland, Australia; PE, Herbarium of Institute of Botany, the Chinese Academy of Sciences, Beijing, China; US, the United States National Herbarium, Washington, D.C. Accession numbers beginning with “KY” are sequences newly generated by this study.*

**Supplementary Table 3. Synonyms for some species of *Tetrastigma* in this study and the related references.**

| **Species** | **Synonyms** | **Reference** |
| --- | --- | --- |
| *Tetrastigma hemsleyanum* | *Tetrastigma bioritsense* | Chen et al., 2007 |
|  | *Tetrastigma alatum* |
|  | *Tetrastigma dentatum* |
| *Tetrastigma henryi* | *Tetrastigma henryi* var. *mollifolium* |
|  | *Tetrastigma lunglingense* |
|  | *Tetrastigma tenue* |
| *Tetrastigma cauliflorum* | *Tetrastigma membranaceum* |
| *Tetrastigma hypoglaucum* | *Tetrastigma obtectum* subsp. *dichotomum* |
|  | *Tetrastigma sinodichotomum* |
| *Tetrastigma serrulatum* | *Tetrastigma napaulense* |
|  | *Tetrastigma indicum* |
| *Tetrastigma obtectum* var. *glabrum* | *Tetrastigma umbellatum* |
| *Tetrastigma obtectum* var*. obtectum* | *Tetrastigma myanmaricum* |
|  | *Tetrastigma obtectum* var*. pilosum* |
| *Tetrastigma pachyphyllum* | *Tetrastigma crassipes* | Chen et al., 2007, Wen et al., 2013 |
|  | *Tetrastigma crassipes* var*. strumarum* |
|  | *Tetrastigma strumarum* |
| *Tetrastigma coriaceum* | *Tetrastigma lanceolarium* | This species is common host plant of *Rafflesia* and has nomenclature confusion. We followed Wen et al. (2013) for this study prior to previously published literature (Latiff, 2001, Veldkamp, 2009) |
|  | *Tetrastigma leucostaphylum* |
|  | *Tetrastigma rafflesia* |
|  | *Tetrastigma tuberculatum* |
| *Tetrastigma diepenhorstii* | *Tetrastigma trifoliolatum* | Latiff, 2001 |

**Supplementary Table 4. Vouchers for the observations of seeds used in this study.**

| **Species** | **Locality** | **Voucher** |
| --- | --- | --- |
| *Causonis japonica* (Thunb.) Raf. | China: Jiangsu | G.X. Fu 93 (PE) |
| *Causonis trifolia* (L.) Raf. | China: Yunnan | Q.W. Wang 80940 (PE) |
| *T. annamense* Gagnep. | Vietnam: Lam Dong | V.C. Dang VN2014119 (PE) |
| *T. campylocarpum* Planch. | China: Yunnan | A. Federov et al.181 (PE) |
| *T. caudatum* Merr. & Chun | China: Guangxi | Chinese and Vietnamese expedition 2143 (PE) |
| *T. cauliflorum* Merr. | China: Hainan | N.Q. Chen 44154 (PE) |
| *T. ceratopetalum* C.Y. Wu | China: Guangxi | Guangxi expedition 3050 (PE) |
| *T. cruciatum* W.G. Craib & Gagnep. | China: Yunnan | Chen et al. DH47 (PE) |
| *T. curtisii* (Ridl.) Suess*.* | Singapore: Bukit Timah | H.N. Ridley s.n. (SING) |
| *T. delavayi* Gagnep. | China: Yunnan | B.Y. Qiu 52815 (PE) |
| *T. ellipticum* Merr. | Philippines: Basilan | J. Reillo 16173 (US) |
| *T. erubescence* Planch. | China: Guangdong | L. Deng 3836 (PE) |
| *T. formosanum* (Hemsl.) Gagnep. | China: Taiwan | C.M. Wang 04412 (PE) |
| *T. harmandii* Planch. | Vietnam | J. Planch. & M.S. Clemens 3992 (US) |
| *T. hemsleyannum* Diels & Gilg | China: Jiangxi | C.M. Tan 971290A (PE) |
| *T. henryi* Gagnep. | China: Yunnan | C. Lu expedition 1405 (PE) |
| *T. hookeri* (M.A. Lawson) Planch. | Vietnam: Kon Tum | D.D. Huyen 604 (HN) |
| *T. hypoglaucum* Planch. ex Franch. | China: Sichuan | Z.W. Yao 2603 (PE) |
| *T. jinghongense* C.L. Li | Myanmar: Putao | Z.D. Chen PT06 (PE) |
| *T. kwangsiense* C.L. Li | China: Guangxi | Nonggan Survey 10957 (IBK) |
| *T. laoticum* Gagnep. | Myanmar: Putao | Z.D. Chen PT03 (PE) |
| *T. lenticellatum* C.Y. Wu | China: Yunnan | Q.W. Wang 79599 (PE) |
| *T. macrocorymbum* Gagnep. ex J. Wen, Boggan & Turland | China: Yunnan | J.S. Xin 233 (PE) |
| *T. nitens* (F. Muell.) Planch. | Australia, Cairns | Lu & Jackes Au 026(PE) |
| *T. obovatum* Gagnep. | China: Yunnan | Chen et al. DH38 (PE) |
| *T. obtectum* (Wall. ex M.A. Lawson) Planch. ex Franch. | China: Hubei | H.J. Li 8739 (PE) |
| *T. pachyphyllum* (Hemsl.) Chun | China: Hainan | Z. Huang 33127 (IBSC) |
| *T. papillosum* Planch. | Philippines | M. Ramos 30348 (US) |
| *T. pedunculare* Planch. | Indonesia: Bukit Bengkirai | Chen & Lu 372 (PE) |
| *T. petraeum* Jackes | Australia | A. Ford 02376 (CNS) |
| *T. pisicarpum* (Miq.) Planch. | Philippines | M. Ramos & S. Fdano 1475 (US) |
| *T. planicaule* (Hook.f.) Gagnep. | China: Fujian | L. Ying 4571 (PE) |
| *T. pseudocruciatum* C.L. Li. | China: Hainan | W.T. Tsang 16430 (PE) |
| *T. pyriforme* Gagnep. | Vietnam: Ha Giang | Harder et al., 5645 (HN) |
| *T. quadrangulum* Gagnep. & Craib | Vietnam: Kon Tum | Bien 646 (HN) |
| *T. retinervum* Planch. | Vietnam: Quang Ninh | Phuong 499 (HN) |
| *T. rumicispermum* (M.A. Lawson) Planch. | China: Tibet | Anonymous 2504 (PE) |
| *T. serrulatum* (Roxb.) Planch. | China: Yunnan | Anonymous 5196 (PE) |
| *T. sichouensis* C.L. Li | Vietnam, Tam Dao | Chen et al. VN0283 (PE) |
| *Tetrastigma* sp.nov. | China | Wen 12026 (unpublished) |
| *T. thorsborneorum* Jackes | Australia, Murray Upper | H. Cook 79 (CNS) |
| *T. tonkinense* Gagnep. | Vietnam: Ninh Binh | Soejarto et al. 12111 (HN) |
| *T. triphyllum* (Gagnep.) W.T. Wang | China: Yunnan | T.N. Liou 14898 (PE) |
| *T. voinierianum* Pierre ex Pit. | Vietnam: Son La | N.T. Cuong & D.D. Cuong VN2067 (HN) |
| *T. xishuangbannaense* C.L. Li | China: Yunnan | G. M. Feng 14486 (PE) |
| *T. yunnannese* Gagnep. | China: Tibet | B.S. Li, S. Cheng 02204 (PE) |

**Supplementary Table 5. Comparison of ancestral states and likelihood proportions for seven key nodes of *Tetrastigma*** based on Mk1 in Mesquite and the best model selected in R (ER or SYM).

|  | Seed shape | | Surface rumination pattern | | Chalaza length/width ratio | | Chalaza position | | Ventral infold position | | Ventral infold divergence | | Ventral infold depth in cross section | | Endosperm shape | |
| --- | --- | --- | --- | --- | --- | --- | --- | --- | --- | --- | --- | --- | --- | --- | --- | --- |
|  | Mk1 | SYM | Mk1 | ER | Mk1 | ER | Mk1 | ER | Mk1 | ER | Mk1 | ER | Mk1 | ER | Mk1 | ER |
| Node 1 | 0 (0.67) | 0 (0.45) | 0 (0.99) | 0 (0.99) | 0 (0.99) | 0 (0.99) | 0 (0.92) | 0(0.96) | 0 (0.80) | 1 (0.56) | 0 (0.84) | 1 (0.47) | 0 (0.55) | 2 (0.86) | 0 (0.65) | 4 (0.68) |
| Node 2 | 1 (1) | 1 (1) | 0 (1) | 0 (1) | 0 (1) | 0 (1) | 0 (1) | 0 (1) | 0 (1) | 0 (1) | 1 (1) | 1 (1) | 1 (1) | 1 (1) | 2 (1) | 2 (1) |
| Node 3 | 3 (0.99) | 3 (0.99) | 0 (0.99) | 0 (0.99) | 0 (0.99) | 0 (0.99) | 2 (0.99) | 2 (0.99) | 1 (0.99) | 1 (0.99) | 0 (0.99) | 0 (0.99) | 2 (0.99) | 2 (0.99) | 2 (0.99) | 2 (0.99) |
| Node 4 | 0 (0.99) | 0 (0.97) | 0 (0.99) | 0 (0.99) | 0 (0.99) | 0 (0.99) | 0 (0.99) | 0 (0.96) | 0 (0.99) | 0 (0.84) | 0 (0.99) | 0 (0.99) | 0 (1) | 0 (1) | 0 (1) | 0 (1) |
| Node 5 | 0 (0.60) | 0 (0.89) | 2 (0.99) | 2 (0.95) | 1 (0.98) | 1 (0.94) | 2 (0.62) | 1 (0.44) | 1 (0.90) | 1 (0.77) | 2 (0.77) | 2 (0.57) | 2 (0.88) | 2 (0.89) | 4 (0.73) | 4 (0.52) |
| Node 6 | 3 (0.92) | 3 (0.87) | 0 (0.99) | 0 (0.99) | 0 (0.99) | 0 (0.99) | 2 (0.85) | 2 (0.73) | 1 (0.99) | 1 (0.99) | 2 (0.99) | 2 (0.99) | 2 (0.99) | 2 (0.99) | 4 (0.99) | 4 (0.99) |
| Node 7 | 0 (0.91) | 0 (0.99) | 0 (0.99) | 0 (0.99) | 0 (0.99) | 0 (0.99) | 0 (0.97) | 0 (0.99) | 0 (0.95) | 0 (0.95) | 0 (0.99) | 0 (0.99) | 0 (0.88) | 0 (0.84) | 0 (0.94) | 0 (0.92) |

*Abbreviations as follows: ER, equal rates; Mk1,* *Markov k-state one-parameter; SYM,* *symmetrical*

**References**

Chen, Z.D., Ren, H., and Wen, J. (2007). “Vitaceae”, in: Wu, Z., Hong, D., Raven, P.H. (Eds.), *Flora of China,* vol. 12. (Science Press and Missouri Botanical Garden Press, Beijing and St. Louis), 173–222.

Fazekas, A.J., Burgess, K.S., Kesanakurti, P.R., Graham, S.W., and Newmaster, S.G. (2008). Multiple Multilocus DNA Barcodes from the Plastid Genome Discriminate Plant Species Equally Well. *PLoS ONE* 3, e2802. doi:10.1371/journal.pone.0002802.

Hollingsworth, M.L., Clark, A.A., Forrest, L.L., Richardson, J., Pennington, R.T., Long, D.G., Cowan, R., Chase, M.W., Gaudeul, M., and Hollingsworth, P.M. (2009). Selecting barcoding loci for plants: evaluation of seven candidate loci with species-level sampling in three divergent groups of land plants. *Mol. Ecol. Resour.* 9, 439–457. doi: 10.1111/j.1755-0998.2008.02439.x

Jackes, B.R. (1989) Revision of the Australian Vitaceae, 5. *Tetrastigma* (Miq.) Planchon. *Austrobaileya* 3, 149–158.

Latiff, A. (2001). Studies in Malesian Vitaceae XII: Taxonomic notes on *Cissus, Ampelocissus, Nothocissus* and *Tetrastigma* and other genera. *Folia Malaysiana* 2, 179–189.

Lee, C., and Wen, J., (2004). Phylogeny of *Panax* using chloroplast *trn*C*–trn*D intergenic region and the utility of *trn*C*–trn*D in interspecific studies of plants. *Mol. Phylogenet. Evol.* 31, 894–903. doi: 10.1016/j.ympev.2003.10.009

Manen, J., Natali, A., and Ehrendorfer, F. (1994). Phylogeny of Rubiaceae-Rubieae inferred from the sequence of a cpDNA intergenic region. *Pl. Syst. Evol.* 190, 195–211. doi: 10.1007/BF00986193

Olmstead, R.G., Michaels, H.J., Scott, K.M., and Palmer J.D. (1992). Monophyly of the Asteridae and identification of their major lineages inferred from DNA sequences of *rbc*L*. Ann. Mo. Bot. Gard.* 79, 249–265.

Ooi, K., Endo, Y., Yokoyama, J., and Murakami, N. (1995). Useful primer designs to amplify DNA fragments of the plastid gene *mat*K from angiosperm plants. *J. Jpn. Bot.* 70, 328–331.

Oxelman, B., Lidén, M., and Berglund, D. (1997). Chloroplast *rps*16 intron phylogeny of the tribe Sileneae (Caryophyllaceae). *Pl. Syst. Evol.* 206, 393–410.

Rossetto, M., Crayn, D.M., Jackes, B.R., and Porter. C. (2007). An updated estimate of intergeneric phylogenetic relationships in the Australian Vitaceae. [*Can. J. Bot*](http://www.cisti.nrc.ca/cisti/journals/tocbot.html)*.* 85, 722–730. doi: 10.1139/B07-022

Sang, T., Crawford, D.J., and Stuessy, T.F. (1997). Chloroplast DNA phylogeny, reticulate evolution, and biogeography of *Paeonia* (Paeoniaceae). *Am. J. Bot.* 84, 1120–1136.

Soejima, A., and Wen, J. (2006). Phylogenetic analysis of the grape family (Vitaceae) based on three chloroplast markers. *Am. J. Bot.* 93, 178–187. doi:10.3732/ajb.93.2.278

Taberlet, P., Gielly, L., Pautou, G., and Bouvet, J. (1991). Universal primers for amplification of three non-coding regions of chloroplast DNA. *Plant Mol. Biol.* 17, 1105–1109.

Veldkamp, J.F. (2009). Notes on the names of the *Tetrastigma* (Vitaceae) hosts of *Rafflesia* (Rafflesiaceae). *Reinwardtia* 13, 75–78.

Wen, J., Lu, L.M., and Boggan, J.K. (2013). Diversity and evolution of Vitaceae in the Philippines. *Philipp. J. Sci.* 142, 223–244.
