# Supplementary material for: Robust Phylogeny of Tetrastigma (Vitaceae) Based on Ten Plastid DNA Regions: Implications for Infrageneric Classification and Seed Character Evolution
Source: Front Plant Sci. 2017 Apr 26;8:590. doi: 10.3389/fpls.2017.00590 (PMC5405133; doi:10.3389/fpls.2017.00590)
Supplement: Supplementary file 2 [file DataSheet2.docx]

# APPENDIX A

**Seed morphological characters and character states of *Tetrastigma***

Character 1 - Seed shape: (0) elliptic, (1) obovoid-elliptic, (2) obovoid, (3) obtriangular (based on Latiff 1983, Li and Wu 1995).

Character 2 - Seed surface rumination pattern: (0) horizontal, (1) smooth, (2) irregular.

Character 3 - Chalaza length/width ratio (dorsal view): (0) ≥ 4, (1) < 4. For chalaza, we initially coded chalaza shape (qualitatively). Later we found this character sometimes difficult to define in discrete states. Thus we code this character quantitatively as chalaza length/width ratio.

Character 4 - Chalaza position (dorsal view): (0) from apex to the base, (1) in the middle, (2) from apex to the middle. These states were consistent with Latiff (1983) and Li and Wu (1995) with slight modification. Chalaza extended from apex to ≥ ¾ of seed length is considered as positioned from apex to base. If the chalaza extended from apex to the middle or slightly below we coded it as apex to the middle.

Character 5 - Ventral infold position (ventral view): (0) entire seed length, (1) ½–¾ of seed length.

Character 6 - Ventral infold divergence (ventral view): (0) diverged from middle or above, (1) parallel, (2) diverged from base. Latiff (1983) regarded ventral infold divergence as a diagnostic character for his section-level classification.

Character 7 - Ventral infold depth in cross section (median cross section view): (0) ¾ of seed length, (1) ≤ ¼ of seed length, (2) ½ of seed length. We found three character states for ventral infold depth in cross section. Ventral infold depth was measured with reference to the seed height in cross section.

Character 8 - Endosperm shape (median cross section view): (0) M-shaped, (1) m-shaped, (2) T-shaped, (3) irregular shaped, (4)
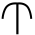
-shaped. M-shaped endosperm is different from the m-shaped endosperm in that the former has ventral infolds inserted > ½ of seed cross section length, whereas the latter has ventral infolds inserted ≤ ¼ of seed cross section length.
